# Radiographic endophenotyping in hip osteoarthritis improves the precision of genetic association analysis

**DOI:** 10.1136/annrheumdis-2016-210373

**Published:** 2017-06-09

**Authors:** Kalliope Panoutsopoulou, Shankar Thiagarajah, Eleni Zengini, Aaron G Day-Williams, Yolande FM Ramos, Jennifer MTA Meessen, Kasper Huetink, Rob GHH Nelissen, Lorraine Southam, N William Rayner, arcOGEN Consortium, Michael Doherty, Ingrid Meulenbelt, Eleftheria Zeggini, J Mark Wilkinson

**Affiliations:** 1 Department of Human Genetics, Wellcome Trust Sanger Institute, Hinxton, UK; 2 Department of Oncology and Metabolism, University of Sheffield, Sheffield, UK; 3 5th Psychiatric Department, Dromokaiteio Psychiatric Hospital of Athens, Athens, Greece; 4 Department of Genomics and Computational Biology, Biogen Idec, Cambridge, Massachusetts, USA; 5 Department of Molecular Epidemiology, LUMC, Leiden, The Netherlands; 6 Department of Orthopaedics, LUMC, Leiden, The Netherlands; 7 Wellcome Trust Centre for Human Genetics, Oxford, UK; 8 Oxford Centre for Diabetes, Endocrinology and Metabolism, University of Oxford, Oxford, UK; 9 Academic Rheumatology, University of Nottingham, Nottingham, UK

**Keywords:** Osteoarthritis, Chondrocytes, Gene Polymorphism

## Abstract

**Objective:**

Osteoarthritis (OA) has a strong genetic component but the success of previous genome-wide association studies (GWAS) has been restricted due to insufficient sample sizes and phenotype heterogeneity. Our aim was to examine the effect of clinically relevant endophenotyping according to site of maximal joint space narrowing (maxJSN) and bone remodelling response on GWAS signal detection in hip OA.

**Methods:**

A stratified GWAS meta-analysis was conducted in 2118 radiographically defined hip OA cases and 6500 population-based controls. Signals were followed up by analysing differential expression of proximal genes for bone remodelling endophenotypes in 33 pairs of macroscopically intact and OA-affected cartilage.

**Results:**

We report suggestive evidence (p<5×10^−6^) of association at 6 variants with OA endophenotypes that would have been missed by using presence of hip OA as the disease end point. For example, in the analysis of hip OA cases with superior maxJSN versus cases with non-superior maxJSN we detected association with a variant in the *LRCH1* gene (rs754106, p=1.49×10^−7^, OR (95% CIs) 0.70 (0.61 to 0.80)). In the comparison of hypertrophic with non-hypertrophic OA the most significant variant was located between *STT3B* and *GADL1* (rs6766414, p=3.13×10^−6^, OR (95% CIs) 1.45 (1.24 to 1.69)). Both of these associations were fully attenuated in non-stratified analyses of all hip OA cases versus population controls (p>0.05). *STT3B* was significantly upregulated in OA-affected versus intact cartilage, particularly in the analysis of hypertrophic and normotrophic compared with atrophic bone remodelling pattern (p=4.2×10^−4^).

**Conclusions:**

Our findings demonstrate that stratification of OA cases into more homogeneous endophenotypes can identify genes of potential functional importance otherwise obscured by disease heterogeneity.

## Introduction

Osteoarthritis (OA) is a phenotypically heterogeneous disease characterised by cartilage degeneration and bone remodelling within synovial joints,^1^ and affects 9 million people in the UK alone.^2^ The heritable component to OA susceptibility is estimated at 40%–65%,^3–5^ however fewer than 20 robustly associated genetic loci have been identified to date explaining <10% of this heritability.^6–18^ These candidate gene and genome-wide association studies (GWAS) used a simple dichotomous description of joint involvement that does not reflect the heterogeneity of OA biology,^19^ with the exception of the *DOT1L* association with hip OA in men that was made by measurement of cartilage thickness.^20^

OA endophenotypes may be characterised on plain radiographs by the pattern of joint involvement and by the bone remodelling response to the disease.^21–23^ At the hip, the site of maximum joint space narrowing (maxJSN) can be classified as superior, medial, axial or concentric.^22^ ^24^ ^25^ The superior pattern is most common in men and is a risk factor for disease progression.^26^ ^27^ Medial migration is more common in women and more likely to occur bilaterally.^26–30^ The bone remodelling response to OA also varies between individuals, and may be classified as atrophic (bony attrition, cysts and a lack of osteophytes), normotrophic (osteophytes <grade 3) or hypertrophic (osteophytes ≥grade 3±increased femoral head size).^23^ Atrophic OA is associated with thinner trabeculae, lower bone volume and lower bone mineral density than the hypertrophic or normotrophic patterns of OA.^31^ ^32^ Atrophic OA is also associated with faster disease progression, osteoporosis and with femoral head collapse.^28^ ^33^ The risk of severe hip OA is threefold greater in siblings of index cases with an atrophic pattern of hip OA compared with siblings whose index case had a normotrophic or hypertrophic bone remodelling response.^27^

Here, we applied these clinically relevant radiographic endophenotypes to identify novel variants that associate with hip OA in a subset of subjects from the arcOGEN Study,^14^ the largest hip and knee OA GWAS to date. We calculated the differences in statistical power achieved using this approach versus a simple dichotomous description of joint involvement. We investigated the effect of using these endophenotype definitions on signal detection for previously established hip OA loci. Finally, we examined the expression levels of genes highlighted in the association analysis in primary human chondrocytes taken from patients classified using the same bone remodelling endophenotypes.

## Patients and methods

### Study populations and radiographic classification

The hip OA subjects comprised 2118 unrelated individuals of UK European ancestry participating in the arcOGEN Study, an ethically approved study in which all subjects provided written, informed consent prior to participation.^14^ All had radiographic evidence of hip OA (Kellgren-Lawrence (KL) Score ≥2) on a digitised anteroposterior radiograph of the affected joint.^21^ Hip OA pattern was classified by the site of maxJSN as superior, axial, medial or concentric using the Osteoarthritis Research Society International (OARSI) atlas and the KL Score (table 1).^21^ ^22^ The bone remodelling response was classified according to the Bombelli and OARSI atlases as atrophic, normotrophic or hypertrophic.^22^ ^23^ Each radiograph was read independently by two clinically trained observers (ST and EZen). Where discrepancy existed between the observers, adjudication was made by the senior clinical author (JMW). The correlation between the two primary observers was ‘very good’ for the axial and medial JSN phenotypes (κ=0.85, 95% CI 0.82 to 0.88)^34^ and ‘good’ for the superior JSN phenotype (κ=0.7, 95% CI 0.67 to 0.73); ‘good’ for the atrophic remodelling phenotype (κ=0.64, 95% CI 0.58 to 0.69) and ‘moderate’ for the hypertrophic phenotype (κ=0.54, 95% CI 0.50 to 0.58).

**Table 1:** Patient characteristics

OA phenotype	arcOGEN_Illum610K N (men/women)	arcOGEN_OmniExpress N (men/women)	N total (men/women)	Prevalence (%)
Hip (KL Score ≥2)	1817 (776/1041)	301 (125/176)	2118 (901/1217)	100
Site of maximal joint space narrowing (pattern of femoral head migration within the acetabulum)				
Axial	158 (34/124)	28 (5/23)	186 (39/147)	8.8
Medial	267 (69/198)	48 (10/38)	315 (79/236)	14.9
Superior	1265 (615/650)	204 (98/106)	1469 (713/756)	69.3
Concentric	127 (58/69)	21(12/9)	148 (70/78)	7.0
Bone remodelling response				
Atrophic	267 (113/154)	18* (8/10)	285 (121/164)	13.4
Hypertrophic	531 (239/292)	106 (46/60)	637 (285/352)	30.1
Normotrophic	1019 (424/595)	177(71/106)	1196(495/701)	56.5

*These individuals were not used as cases in the within-OA genome-wide association analysis of the arcOGEN OmniExpress data set due to the small sample number.

KL, Kellgren-Lawrence; OA, osteoarthritis.

### Genotyping, imputation and association analyses

**Figure 1:**
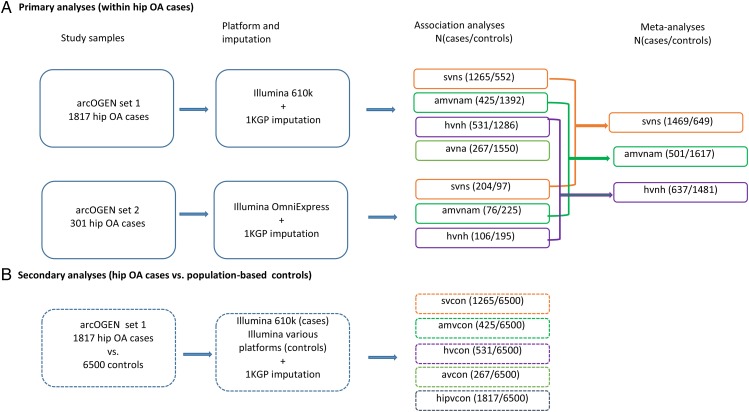
Genome-wide association study design, showing primary and secondary analyses. svns, superior joint space narrowing versus non-superior; amvnam, axial/medial joint space narrowing versus non-axial/medial; hvnh, hypertrophic bone response versus non-hypertrophic; avna, atrophic bone response versus non-atrophic; svcon, superior joint space narrowing versus population-based controls; amvcon, axial/medial joint space narrowing versus population-based controls; hvcon, hypertrophic bone response versus population-based controls; avcon, atrophic bone response versus population-based controls; hipvcon, hip osteoarthritis (OA) versus population-based controls.

Genotypes were extracted from 1817 and 301 hip OA cases previously genotyped on Human 610-Quad and HumanOmniExpress (Illumina, San Diego, USA) platforms, respectively (figure 1). The controls comprised 6500 subjects previously included in the arcOGEN GWAS: The Wellcome Trust Case Control Consortium 2 Study 1958 Birth Cohort, the UK National Blood Donor Service and the Type 1 Diabetes Genetics Consortium.^14^ A 1000 Genomes Project-based imputation was conducted,^35^ ^36^ and was followed by stringent quality control excluding variants with minor allele frequency (MAF)<0.01 and minor allele count <10 given the different sample sizes of the GWAS case cohorts,^37^ imputation information score <0.4 and by inspecting genotype intensity plots 100 kb on either side of the top signals and re-imputation of these regions of association.

Association analyses at ∼7 million variants were made under an additive model (maximum likelihood ratio test, SNPTESTv2.5). Our primary analyses comprised fixed-effect meta-analyses^38^ within OA cases stratified by site of maxJSN or by bone remodelling response to identify variants exclusively associated with these endophenotypes (table 1 and figure 1). The axial and medial maxJSN groups were collapsed into one (axial/medial) due to the small sample size in each of these categories. Promising signals were independently validated by de novo genotyping using the MassARRAY iPLEX Gold assay (Agena Bioscience GmbH, Hamburg, Germany).

In our secondary analyses the GWAS outputs for the stratified hip OA cases against population-based controls were compared with that achieved using a non-stratified GWAS of all hip OA cases versus population-based controls. Power was calculated using Quanto V.1.2.4 under the additive model^39^ at α=5×10^−8^, and based on study-specific effect sizes and case-control ratios identified in the Human 610-Quad GWAS. Similarly, the sample size required to have 80% power to detect the associated signals with genome-wide significance (p=5×10^−8^) was estimated. To calculate the sample size fold reduction afforded by the use of precise phenotype definitions over an all hip OA versus controls approach we fixed the estimated sample size of hip OA cases from infinite to n=1 000 000.

### Gene expression analysis

Gene expression profiles were examined in samples from the RAAK Study (Research Arthritis and Articular Cartilage) from individuals undergoing joint replacement surgery, as described previously.^40^ Isolated RNA was prepared for hybridisation using the Illumina TotalPrep RNA Amplification Kit (Illumina, San Diego, USA) onto Illumina HumanHT-12 v3 microarrays, with each pair (OA-affected and intact) measured on the same chip. Microarrays were read using an Illumina Beadarray 500GX scanner and data were analysed in R statistical programming language after quality checks using Illumina Beadstudio analysis software (Illumina). Intensity values were normalised and absence of large-scale between-chip effects was confirmed using the Globaltest-package.^41^ Probes that were not optimally measured were removed (detection p>0.05 in more than 50% of the samples). All comparisons of the differential normalised probe expression levels between intact and diseased OA chondrocytes were made in SPSS (V.23, IBM, New York, USA) using the generalised estimation equations and corrected for age and sex. A random effect for patient identity (ID) was included to account for the structure of the data and correct for putative correlations between intact and OA-affected articular cartilage from the same joint.

Preoperative plain radiographs of the affected joint were scored independently by two experienced readers (JMTAM, KH) to determine the OA subtype. Subsequently, osteophytes and JSN were graded 0–3 for lateral and medial femur and tibia (knee joints) and for inferior and superior femur and acetabulum (hip joints) by consensus opinion using the OARSI atlas.^22^

## Results

### Site of maxJSN association analysis

In the GWAS meta-analysis of hip OA cases with superior maxJSN versus all other hip OA cases (see online supplementary figure S1a), the most significant association was observed at rs754106 (MAF=0.46) in intron 1 of the *LRCH1* (leucine-rich repeats and calponin homology domain containing 1) gene (T allele OR (95% CIs) 0.70 (0.61 to 0.80), p=1.49×10^−7^ (table 2 and see online supplementary figure S2a)), and was consistent across both cohorts (see online supplementary table S1). In the GWAS meta-analysis of hip OA cases with axial/medial maxJSN versus all other hip OA cases (see online supplementary figure S1b), rs754106 was also the most significantly associated variant (p=3.66×10^-7^) but with the effect of the T allele in the opposite direction, in keeping with expectation (OR (95% CIs) 1.47 (1.27 to 1.70), table 2 and see online supplementary table S1).

**Table 2:** Meta-analysis summary statistics for the most significantly associated variants with maximal site of JSN and with bone remodelling response phenotypes of hip OA

Study characteristics			SNP characteristics						Meta-analysis		Sex-adjusted meta-analysis	
Cases definition	Controls definition	N case/control	SNP	Chr	Position (b37)	Nearest gene(s)	EA	EAF case/control	OR (95% CI)	p Value	OR (95% CI)	p Value
Hip OA with superior JSN	Hip OA with non-superior JSN	1469/649	rs754106	13	47 152 655	*LRCH1*	T	0.51/0.60	0.70 (0.61 to 0.80)	1.49×10^−7^	0.72 (0.62 to 0.82)	2.17×10^−6^
Hip OA with axial/medial JSN	Hip OA with non-axial/medial JSN	501/1617	rs754106	13	47 152 655	*LRCH1*	T	0.61/0.52	1.47 (1.27 to 1.70)	3.66×10^−7^	1.42 (1.22 to 1.65)	6.92×10^−6^
Hip OA with superior JSN	Hip OA with non-superior JSN	1469/649	rs73023563	2	181 502 830	*CWC22* *UBE2E3*	T	0.21/0.14	1.59 (1.33 to 1.91)	6.59×10^−7^	1.58 (1.31 to 1.90)	1.65×10^−6^
Hip OA with axial/medial JSN	Hip OA with non-axial/medial JSN	501/1617	rs73023563	2	181 502 830	*CWC22* *UBE2E3*	T	0.14/0.20	0.61 (0.50 to 0.75)	2.03×10^−6^	0.62 (0.50 to 0.76)	5.44×10^−6^
Hip OA with axial/medial JSN	Hip OA with non-axial/medial JSN	501/1617	rs17050727	4	121 426 827	*PRDM5*	T	0.18/0.12	1.65 (1.35 to 2.01)	6.87×10^−7^	1.67 (1.36 to 2.05)	8.38×10^−7^
Hip OA with hypertrophic response	Hip OA with non-hypertrophic response	637/1481	rs6766414	3	31 488 222	*STT3B* *GADL1*	G	0.29/0.22	1.45 (1.24 to 1.69)	3.13×10^−6^	1.46 (1.25 to 1.70)	2.31×10^−6^
Hip OA with hypertrophic response	Hip OA with non-hypertrophic response	637/1481	rs61837881	1	223 626 562	*CAPN8* *C1orf65*	C	0.29/0.22	1.44 (1.23 to 1.68)	4.56×10^−6^	1.43 (1.23 to 1.67)	5.55×10^−6^

EA, effect allele; EAF, effect allele frequency; JSN, joint space narrowing; OA, osteoarthritis; SNP, single nucleotide polymorphism.

10.1136/annrheumdis-2016-210373.supp1 supplementary data

In the association analyses of hip OA cases with superior maxJSN versus population-based controls (see online supplementary figure S3a) and of patients with hip OA with axial/medial maxJSN versus population-based controls (see online supplementary figure S3b), opposite directions of effect were also observed for allele T: OR (95% CIs) 0.91 (0.84 to 0.99), p=0.034 compared with OR (95% CIs) 1.32 (1.14 to 1.52), p=1.39×10^−4^, respectively (see online supplementary table S1). When all hip OA cases were analysed together versus the same population-based control set, the signal was fully attenuated (OR (95% CIs) 1.01 (0.93 to 1.08), p=0.89) (see online supplementary table S1 and figure S3e).

The second most significant finding in the superior maxJSN stratum versus all other hip OA cases was observed at rs73023563 (MAF=0.19) located between *CWC22* (spliceosome-associated protein homologue (S. Cerevisiae)) and *UBE2E3* (ubiquitin-conjugating enzyme E2E 3) (T allele OR (95% CIs) 1.59 (1.33 to 1.91), p=6.59×10^−7^, table 2 and see online supplementary figure S2b). The rs73023563 association was also strong in the converse stratum (hip OA cases with axial/medial maxJSN vs all other hip OA cases) with the opposite direction of effect (T allele OR (95% CIs) 0.61 (0.50 to 0.75), p=2.03×10^−6^). Its association in the analysis of axial/medial maxJSN cases versus population-based controls was equally strong (OR (95% CIs) 0.63 (0.52 to 0.78), p=3.45×10^−6^), and was also fully attenuated in the all hip OA analysis versus population-based controls analysis (OR (95% CIs) 0.94 (0.86 to 1.03), p=0.21) (see online supplementary table S1).

In the association analysis of hip OA with axial/medial maxJSN versus all other hip OA cases the second most significant single nucleotide polymorphism (SNP) was rs17050727 (MAF=0.14), located between *PRDM5* (PR domain containing 5) and *MAD2L1* (mitotic arrest deficient-like 1 (yeast), T allele OR (95% CIs) 1.65 (1.35 to 2.01), p=6.87×10^−7^ (table 2, see online supplementary table S1 and figure S2c). rs17050727 exhibited a similar pattern of association with the converse stratum (superior maxJSN vs all other hip OA cases) as for the variants described above; and its association was also fully attenuated in the hip OA analysis versus population-based controls analysis (OR (95% CIs) 1.00 (0.90 to 1.12), p=0.98, see online supplementary table S1).

Adjustments for sex, height and body mass index (BMI) had little effect on the association strength of the reported signals (see online supplementary table S2). Following validation experiments by de novo genotyping we found 100% genotype and minor allele concordance with GWAS genotypes of overlapping samples at rs754106 (see online supplementary table S3).

### Bone remodelling response association analysis

In the GWAS meta-analysis of hypertrophic versus no-hypertrophic hip OA cases (see online supplementary figure S1c) the most significant SNP was rs6766414 (MAF=0.24) located between *STT3B* (subunit of the oligosaccharyltransferase complex (catalytic)) and *GADL1* (glutamate decarboxylase-like 1), G allele OR (95% CIs) 1.45 (1.24 to 1.69), p=3.13×10^−6^ (table 1 and see online supplementary figure S2d). We also found evidence of association in the hypertrophic OA versus population-based controls analysis, G allele OR (95% CIs) 1.25 (1.09 to 1.44), p=1.74×10^−3^ (see online supplementary table S1 and figure S3c). In the analysis of hip OA cases versus population-based controls the signal was fully attenuated (OR (95% CIs) 1.00 (0.92 to 1.09), p=0.98) (see online supplementary table S1).

The second most significant replicating SNP in the hypertrophic versus non-hypertrophic stratum, rs61837881 (MAF=0.24) located between *C1orf65* and *CAPN8* (calpain 8), was associated exclusively with the hypertrophic response (allele C: OR (95% CIs) 1.44 (1.23 to 1.68), p=4.56×10^−6^, see online supplementary figure S2e) and was fully attenuated in the analysis of hip OA versus population-based controls (OR (95% CIs) 1.06 (0.97 to 1.15), p=0.23, table 2 and see online supplementary table S1). Adjustment for sex, height and BMI had little effect on the association strength of the reported variants (table 2 and see online supplementary table S2).

In the GWAS of atrophic versus non-atrophic OA (see online supplementary figure S1d), the most significant association was observed at rs16869403, which resides within the gene encoding the G protein-coupled receptor 98 (*GPR98*) (MAF=0.10, allele G OR (95% CI) 2.11 (1.61 to 2.75), p=1.37×10^−7^, see online supplementary figure S2f and table S1). The strength of association was similar in the analysis of atrophic OA cases versus population-based controls (OR (95% CI) 1.93 (1.52 to 2.46), p=4.14×10^−7^, see online supplementary table S1 and figure S3d). This association was fully attenuated in the non-stratified hip OA versus population-based controls analysis (OR (95% CI) 1.05 (0.93 to 1.19), p=0.43; see online supplementary table S1). Genotype and minor allele concordance between typed and imputed genotypes at this SNP were 99% and 97%, respectively (see online supplementary table S3).

### Power gains

Power increases substantially in the within-OA analyses compared with the non-stratified hip OA versus population-based controls analysis (table 3). Small to modest power increases are observed in the stratified OA cases versus population-based controls analyses compared with the non-stratified hip OA versus population-based controls analysis. The decrease in sample size required to reach genome-wide significance for an analysis within OA cases over the non-stratified hip OA versus population-based controls analysis ranged from 41-fold to 286-fold. The decrease in sample size between stratified hip OA versus population-based controls analyses over the non-stratified hip OA versus population-based controls analysis ranged from 1-fold to >240-fold.

**Table 3:** Power gains associated with dichotomous versus specific radiographic endophenotype descriptors

Study parameters							Estimated parameters			
SNP	Definition of cases	Definition of controls	N cases	N controls	EAF	OR	Power (%)	N cases	N controls	N total
rs754106	Hip OA with superior JSN	Hip OA with non-superior JSN	1265	552	0.54	0.72	18.45	2416	1054	3470
	Hip OA with superior JSN	Population-based	1265	6500	0.54	0.91	0.05	10 669	54 821	65 490
	Hip OA	Population-based	1817	6500	0.54	1.01	0.00	>1 000 000	>3 577 325	>4 577 325
rs73023563	Hip OA with superior JSN	Hip OA with non-superior JSN	1265	552	0.19	1.63	57.78	1571	686	2257
	Hip OA with superior JSN	Population-based	1265	6500	0.20	1.07	0.00	31 513	161 925	193 438
	Hip OA	Population-based	1817	6500	0.19	0.94	0.00	43 995	157 384	201 379
rs17050727	Hip OA with axial/medial JSN	Hip OA with non-axial/medial JSN	425	1392	0.14	1.65	28.62	705	2309	3014
	Hip OA with axial/medial JSN	Population-based	425	6500	0.14	1.43	4.89	1168	17 864	19 032
	Hip OA	Population-based	1817	6500	0.14	1.00	0.00	∞	∞	∞
rs6766414	Hip OA with hypertrophic response	Hip OA with non-hypertrophic response	531	1286	0.24	1.42	12.40	1139	2758	3897
	Hip OA with hypertrophic response	Population-based	531	6500	0.24	1.25	0.92	2199	26 918	29 117
	Hip OA	Population-based	1817	6500	0.24	1.00	0.00	∞	∞	∞
rs61837881	Hip OA with hypertrophic response	Hip OA with non-hypertrophic response	531	1286	0.24	1.39	7.69	1298	3144	4442
	Hip OA with hypertrophic response	Population-based	531	6500	0.24	1.33	7.15	1323	16 195	17 518
	Hip OA	Population-based	1817	6500	0.24	1.06	0.00	40 187	143 762	183 949
rs16869403	Hip OA with atrophic response	Hip OA with non-atrophic response	267	1550	0.10	2.11	58.36	330	1916	2246
	Hip OA with atrophic response	Population-based	267	6500	0.10	1.93	42.25	383	9324	9707
	Hip OA	Population-based	1817	6500	0.10	1.05	0.00	115 540	413 324	528 864

For each variant and stratum we present power (%) based on study-specific effect size, effect allele frequency and case/control ratio from the GWAS on Illumina610k. Similarly we estimated the sample size required to have 80% power to detect the associated signals with genome-wide significance (p=5×10^−8^).

EAF, effect allele frequency; GWAS, genome-wide association studies; JSN, joint space narrowing; OA, osteoarthritis; SNP, single nucleotide polymorphism.

### Established hip OA loci

The strength of association of the 10 established hip OA susceptibility loci in Europeans^18^ was examined in each of the hip OA strata (see online supplementary table S4). Here, the risk allele of rs12982744 in *DOTL1* associated with hip OA (p=3.06×10^−4^, OR (95% CIs) 1.15 (1.07 to 1.24)) but the most significant association was observed in the analysis of hip OA with superior maxJSN versus population-based controls (p=8.17×10^−6^, OR (95% CIs) 1.22 (1.12 to 1.34)) (see online supplementary table S4). Further corroboration of this stratum-specific association comes from the within-OA GWAS analyses of the hip OA cases with superior maxJSN versus all other hip OA cases (p=7.58×10^−3^, OR (95% CIs) 1.22 (1.06 to 1.42); and converse stratum p=1.13×10^−2^, OR (95% CIs) 0.81 (0.69 to 0.95)). The effect size differed between men and women both in our hip OA versus population-based control analysis (men p=8.92×10^−6^, OR (95% CIs) 1.30 (1.16 to 1.46); women p=0.32, OR (95% CIs) 1.05 (0.95 to 1.17)), and in the superior maxJSN versus population-based controls analysis (men p=5.20×10^−6^, OR (95% CIs) 1.35 (1.18 to 1.53); women p=0.07, OR (95% CIs) 1.12 (0.99 to 1.27)). This suggests that the sex disparity of effects at the *DOT1L* locus is not driven by the differences in prevalence of different JSN patterns between the two sexes.

Five other index SNPs (rs9350591, rs10948172, rs11177, rs4836732, rs6094710) also exhibited similar or lower p values and higher ORs in endophenotype-based association analyses than in the hip OA versus population-based controls analysis, despite the decrease in case sample size (ranging from 30% to 77%) (see online supplementary table S4). However, unlike the *DOT1L* variant, none of these showed an association that was strengthened enough to compensate for the multiple comparisons performed.

### Gene expression analyses in articular cartilage

**Figure 2:**
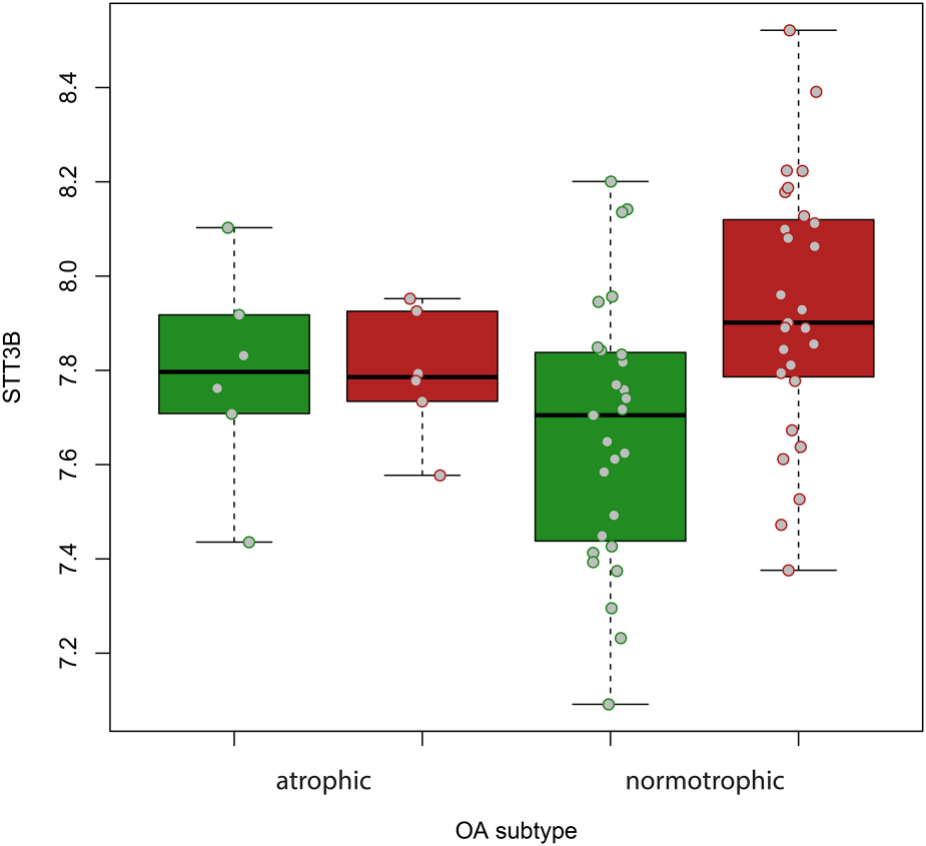
Box plots of the expression levels of *STT3B* plotted as the mean normalised probe level values in macroscopically intact (green boxes) and diseased (red boxes) osteoarthritis (OA) articular cartilage of patients with atrophic (n=6) and normotrophic (n=27) OA. Grey dots show values of each individual sample.

**Figure 3:**
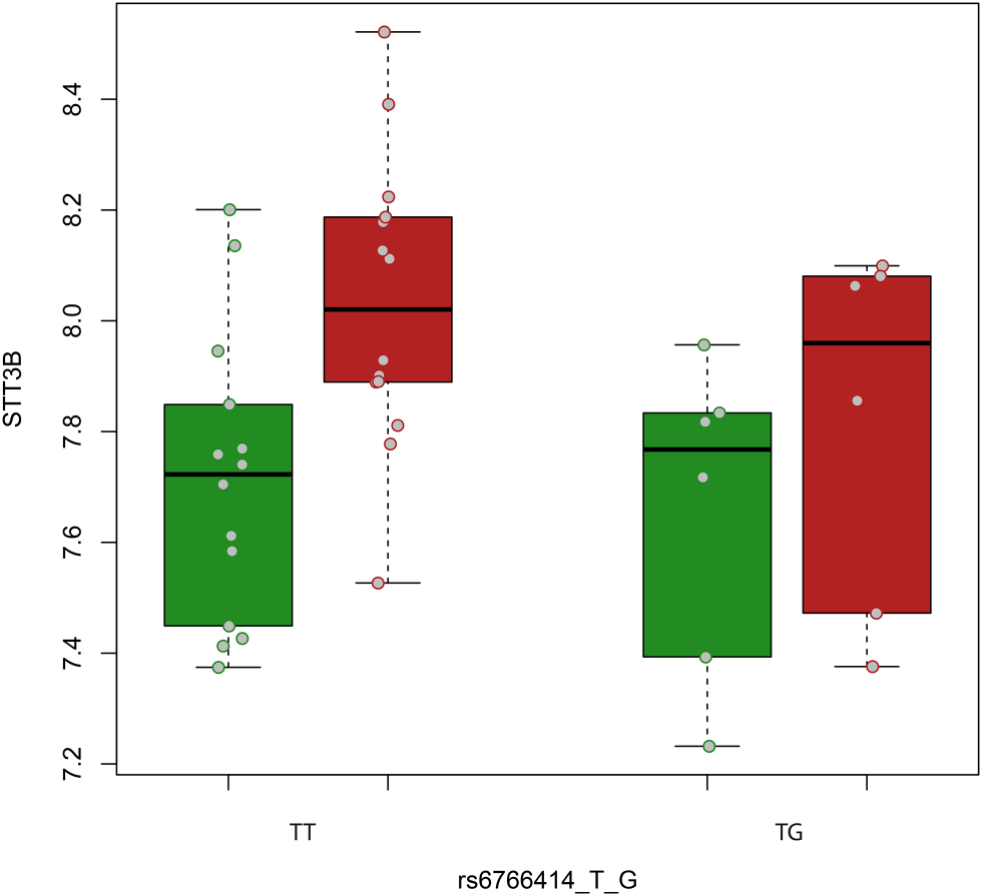
Box plots of the expression levels of *STT3B* in patients with hypertrophic/normotrophic osteoarthritis (OA), plotted as mean normalised probe level values, in macroscopically intact (green boxes) and diseased (red boxes) OA articular cartilage stratified by carriers of the G allele (heterozygous individuals plotted since no homozygotes for the G allele were present in our data) and by non-carriers at rs6766414. Grey dots show values of each individual sample.

The expression levels of 10 genes located near variants highlighted in the bone remodelling stratified GWAS (*GRP98* and *ADGVR1* for rs16869403; *STT3B*, *GADL1*, *OSBPL10*, *THRAP3* and *TGFBR2* for rs6766414; and *C1orf65*, *CAPN8* and *SUSD4* for rs61837881) were examined in macroscopically intact and OA-affected articular cartilage (n=33 pairs from the RAAK Study, 6 with an atrophic, 19 with a normotrophic and 8 with a hypertrophic phenotype). We observed robust expression for *SUSD4*, *STT3B*, *OSBPL10* and *TGFBR2*, with mean normalised probe level values of 7.9, 7.8, 8.4 and 11.5, respectively. Of these genes, only *STT3B* showed differential expression with respect to the disease process, with significant upregulation in OA-affected versus intact cartilage independent of joint site, particularly in patients with hypertrophic/normotrophic OA (fold change=1.30, p=4.23×10^−4^; figure 2). When the genotypes of rs6766414 (TT and TG) were tested, a significantly lower expression of *STT3B* in lesioned OA cartilage was observed among carriers of allele G (n=9 heterozygotes-no homozygotes for the G allele were present in our data) compared with homozygous individuals for allele T (n=15; fold change=0.79, p=0.021). Moreover, in the hypertrophic/normotrophic OA stratum, the upregulation of *STT3B* expression in OA-affected versus intact cartilage appeared to be mitigated among individuals heterozygous for allele G of rs6766414 (n=6), yet remained significantly upregulated among homozygous carriers (n=14) of allele T (fold change=1.4, p=2.8×10^−4^, figure 3).

## Discussion

We applied clinical radiographic endophenotypes to stratify genome-wide association analysis of hip OA and identify six putative associations (p<5×10^−6^). For the strongest signals in both endophenotypes we observe consistent effects across complementary strata both in the primary analysis and against population-based controls in the secondary analyses. These signals were all fully attenuated in the non-stratified analyses against the population-based controls, consistent with our hypothesis that this stratification approach increases the sensitivity of signal detection.

rs754106, which showed the strongest association with pattern of maxJSN, resides in intron 1 of the *LRCH1/CHDC1* gene whose function has not been well characterised. However, replicating associations with knee OA at variants spanning intron 1 of *LRCH1* have been identified in samples of European descent.^42^ rs6766414, which showed the strongest association with bone remodelling response, is located between *STT3B* and *GADL1. STT3B* mediates both co-translational and post-translational N-glycosylation of proteins and GO terms associated with this gene include glycoprotein catabolic process.^43^ *STT3B* encodes a catalytic subunit of the N-oligosaccaryl transferase complex,^44^ suggesting that altered glycosylation in OA-affected cartilage mitigates protein folding and affects intermolecular interactions.^45^ Thus we hypothesise that the upregulation of *STT3B* is a beneficial response among hypertrophic cases.

This study also has limitations. While the interobserver agreement of the radiographic classifiers supports the approach taken, differences in calling between observers does impact on the overall sensitivity of both the genetic association and expression analyses. Although the identified loci did not meet the Bonferroni-adjusted genome-wide significance threshold of p<5×10^−9^ used because of the multiple discovery association analyses conducted, the putative association of variants with specific endophenotypes corroborated by substantial or full attenuation in other strata warrants replication and further functional investigation.

The hypothesis that stratification of cases into distinct endophenotypic categories provides a more direct link to the genetic basis of OA has not been previously tested systematically. However, this approach has been successfully applied to better understand disease biology in related complex traits, including joint shape and pain.^46–50^ The results of our analyses show that, if further replication is targeted to the specific OA endophenotypes, study power may improve from nearly 0% in the traditional approach to 80% in a sample size of <5000 cases. We used post hoc power calculations, applying the observed variances from the study population of interest to exemplify the difference in the statistical power of the tests we performed. Such calculations have limitations, but are useful in designing follow-up studies and in conducting meta-analyses. We address one limitation in interpreting post hoc power calculations, ascertainment bias, by examining the actual summary statistics of the previously established genome-wide significant locus (*DOT1L*) associated with JSN. We find that the index SNP is more strongly associated in hip OA cases with superior JSN than in the non-stratified hip OA versus controls analyses (p=8×10^−6^ and p=3×10^−4^, respectively). As more precise phenotype definitions are accrued in the field and future replication is targeted to the specific endophenotype, the strength of the observed associations may increase and the power burden of multiple testing may be resolved. Our findings demonstrate that stratification of OA cases into more homogeneous endophenotypes can identify genes of potential functional importance otherwise obscured by disease heterogeneity.
