# Supplementary material for: Radiographic endophenotyping in hip osteoarthritis improves the precision of genetic association analysis
Source: Ann Rheum Dis. 2017 Jun 9;76(7):1199–206. doi: 10.1136/annrheumdis-2016-210373 (PMC5530347; doi:10.1136/annrheumdis-2016-210373)
Supplement: supplementary data [file annrheumdis-2016-210373supp001.pdf]

SUPPLEMENTARY MATERIAL

SUPPLEMENTARY FIGURES

Figure S1. QQ and Manhattan plots for primary within OA case/control analyses of hip OA stratified by anatomic pattern of joint involvement and by bone remodeling response. (a) GWAS meta-analysis of hip OA cases with superior joint space narrowing vs. hip OA cases with axial, medial and concentric pattern; (b) GWAS meta-analysis of hip OA cases with axial/medial joint space narrowing vs. hip OA cases with superior and concentric pattern; (c) GWAS meta-analysis of hip OA cases with hypertrophic bone response vs. hip OA cases with atrophic and normotrophic bone response; (d) GWAS of hip OA cases with atrophic bone response vs. hip OA cases with hypertrophic and normotrophic bone response.

(a)

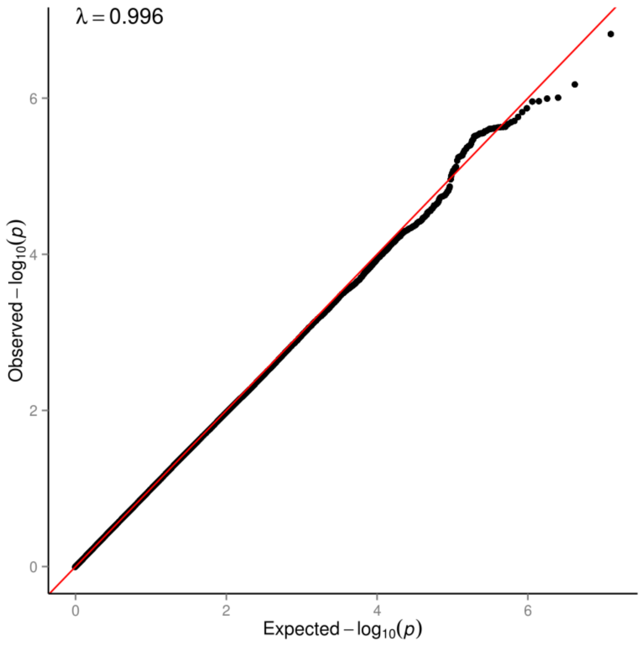

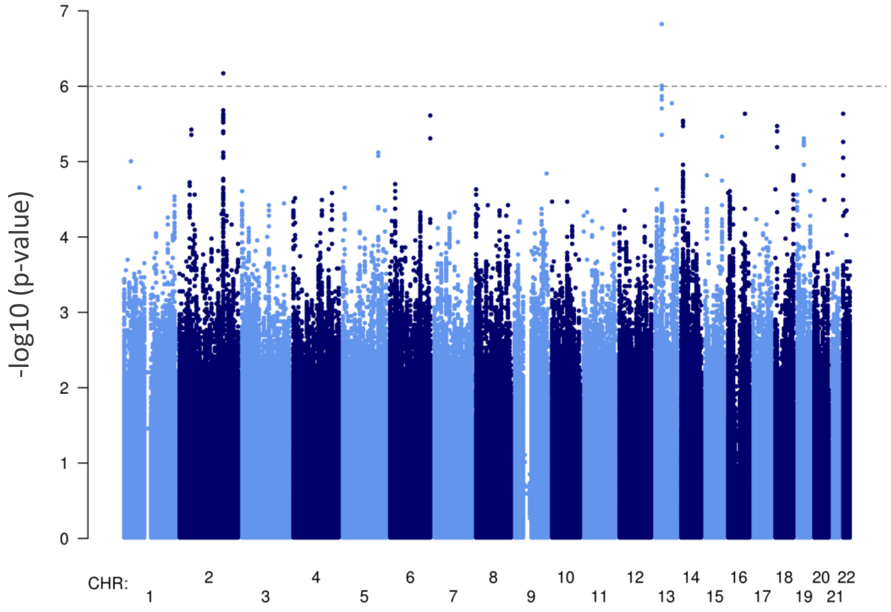
-log10 (p-value)
CHR:
1 2 3 4 5 6 7 8 9 10 11 12 13 14 15 16 17 18 19 20 21 22

(b)

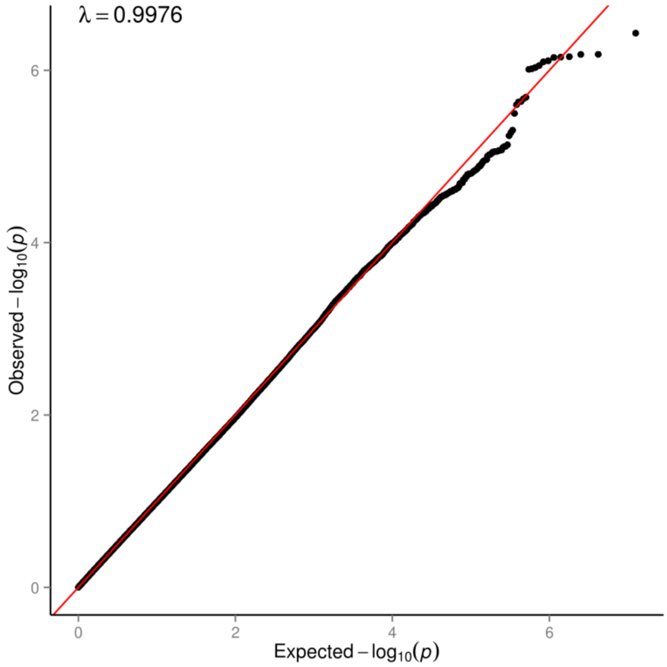
λ = 0.9976
Observed − log10(p)
Expected − log10(p)

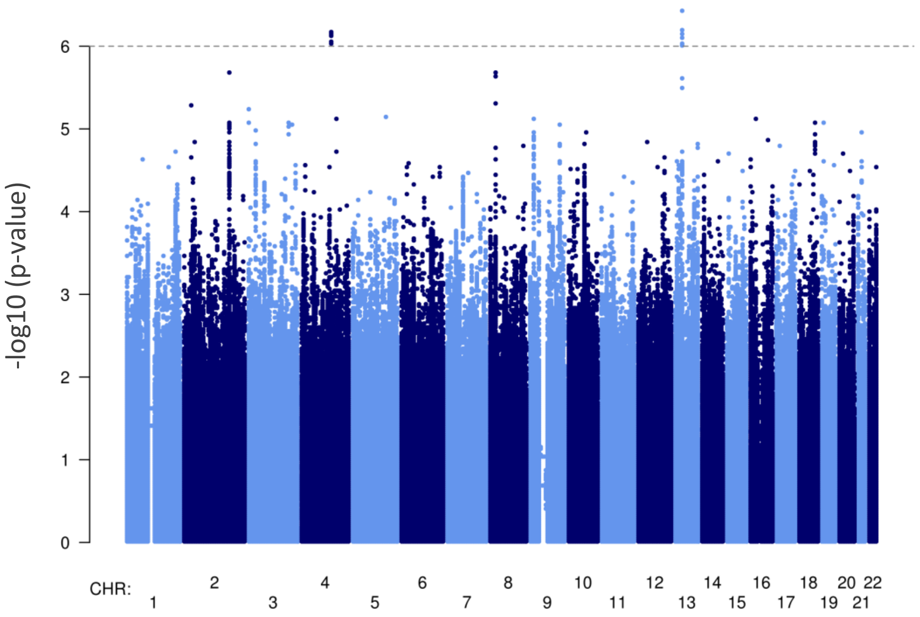

-log10 (p-value)
CHR:
1
2
3
4
5
6
7
8
9
10
11
12
13
14
15
16
17
18
19
20
21
22

(c)

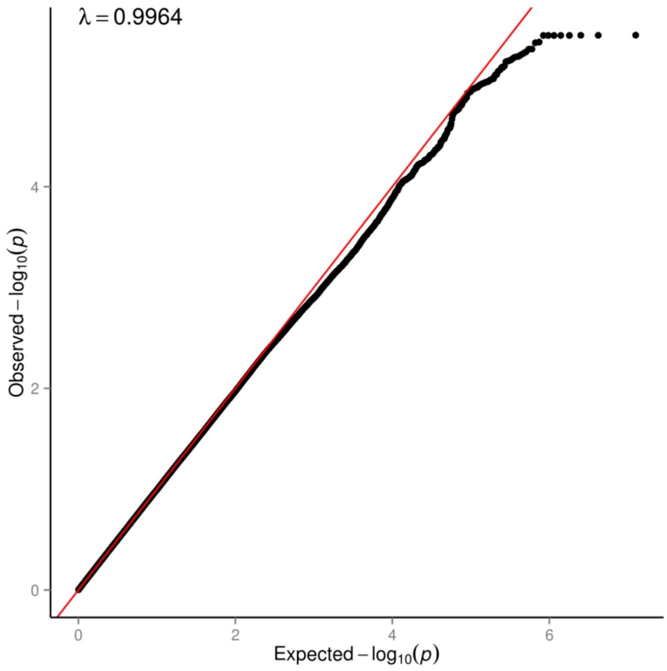

λ = 0.9964
Observed − log10(p)
Expected − log10(p)

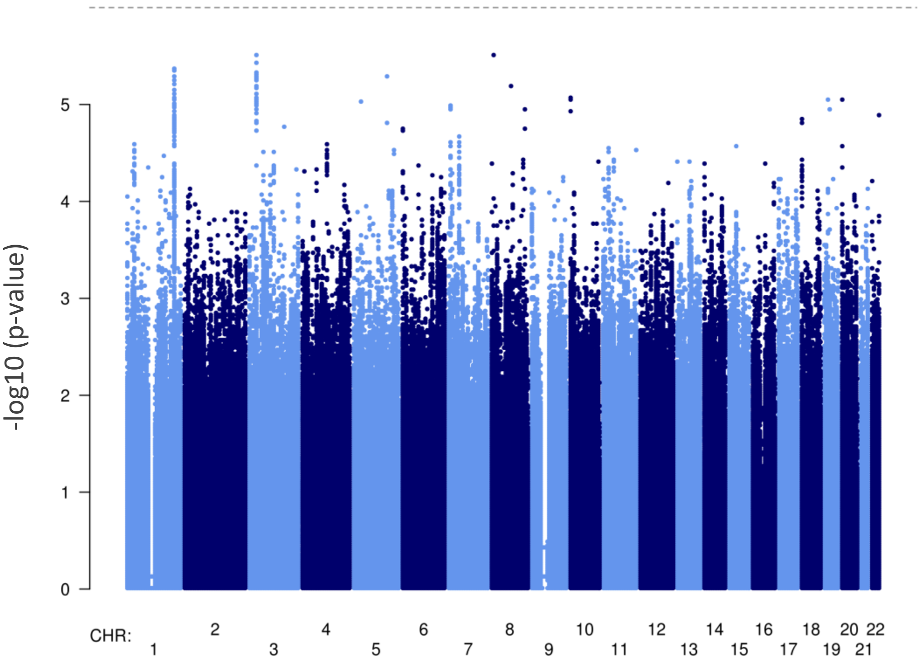
-log10 (p-value)
0
1
2
3
4
5
CHR:
1 2 3 4 5 6 7 8 9 10 11 12 13 14 15 16 17 18 19 20 21 22

(d)

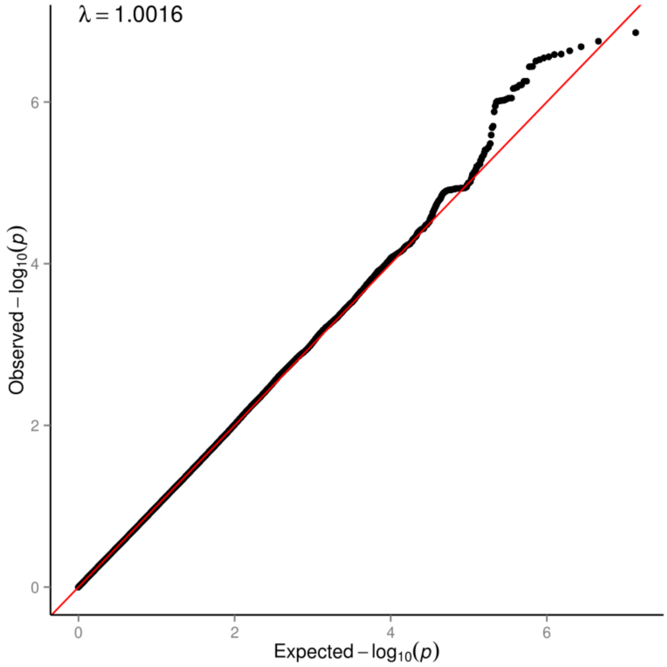
λ = 1.0016
Observed − log10(p)
Expected − log10(p)
0
2
4
6

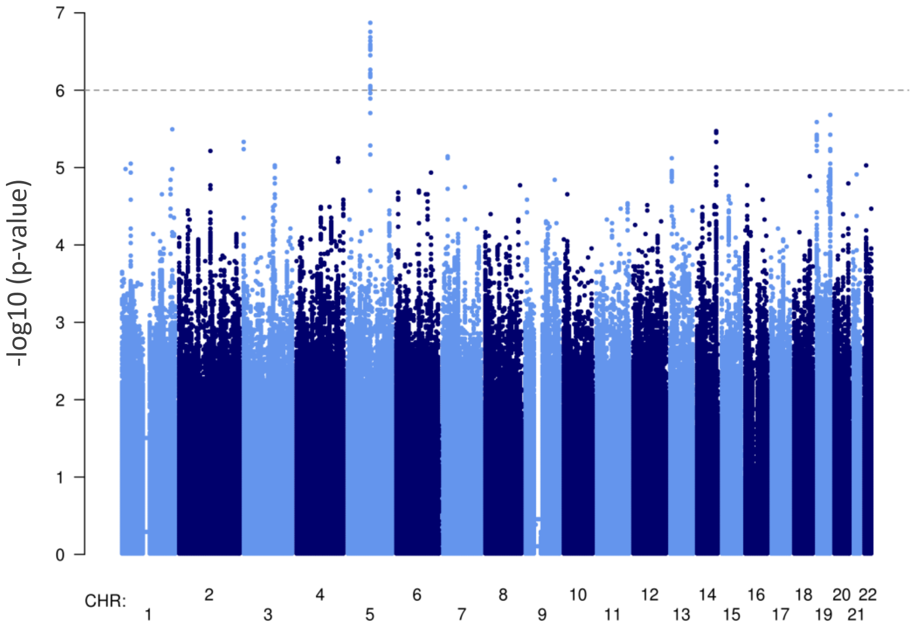

Figure S2. Regional association plots for top signals.

The purple dot denotes the p-value of the index variant and the colour of the other dots shows the pairwise linkage disequilibrium (r^2) with neighbouring variants in the region of association based on the 1000 Genomes EUR panel. (a) Association at the rs754106 locus in the GWAS meta-analysis of hip OA cases with superior joint space narrowing vs. cases with non-superior joint space narrowing. (b) Association at the rs73023563 locus in the GWAS meta-analysis of hip OA cases with superior joint space narrowing vs. cases with non-superior joint space narrowing. (c) Association at the rs17050727 locus in the GWAS meta-analysis of hip OA cases with axial/medial joint space narrowing vs. cases with non-axial/medial joint space narrowing. (d) Association at the rs6766414 locus in the GWAS meta-analysis of hip OA cases with hypertrophic bone response vs. non-hypertrophic response. (e) Association at the rs61837881 locus in the GWAS meta-analysis of hip OA cases with hypertrophic bone response vs. non-hypertrophic response. (f) Association at the rs61837881 locus in the GWAS of hip OA cases with atrophic bone response vs. non-atrophic hip OA cases.

(a)

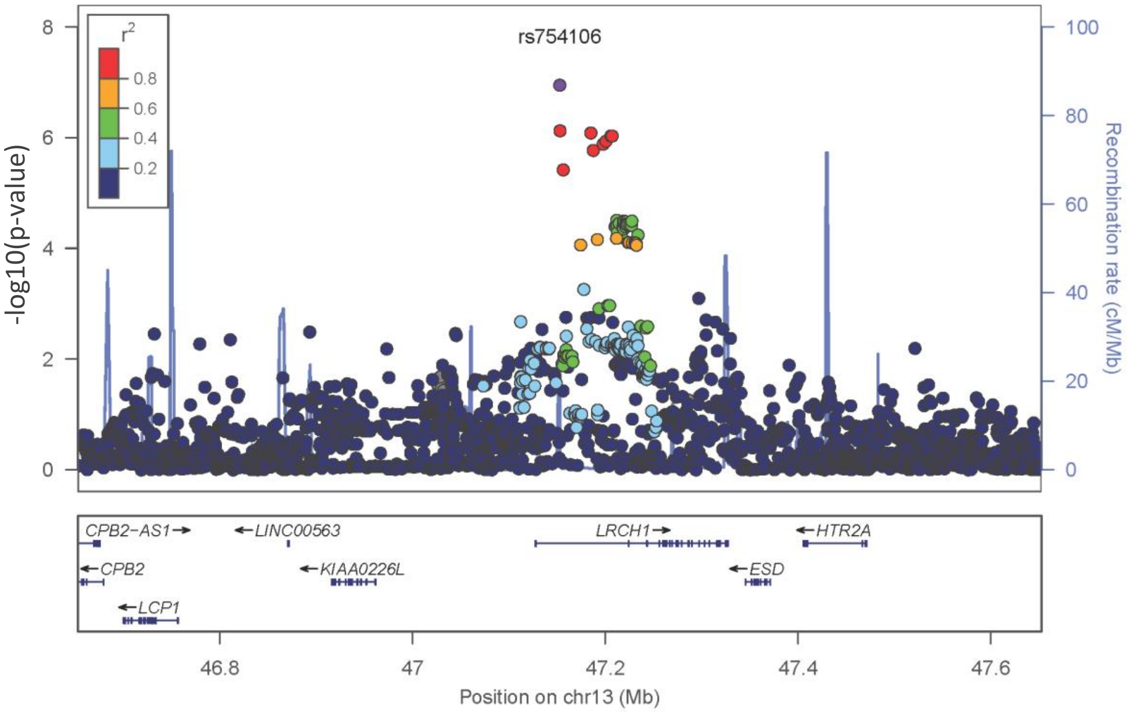

rs754106
-log10(p-value)
Recombination rate (cM/Mb)
r2
0.8
0.6
0.4
0.2
8
6
4
2
0
100
80
60
40
20
0
CPB2−AS1
LINC00563
LRCH1
HTR2A
CPB2
KIAA0226L
ESD
LCP1
46.8
47
47.2
47.4
47.6
Position on chr13 (Mb)

(b)

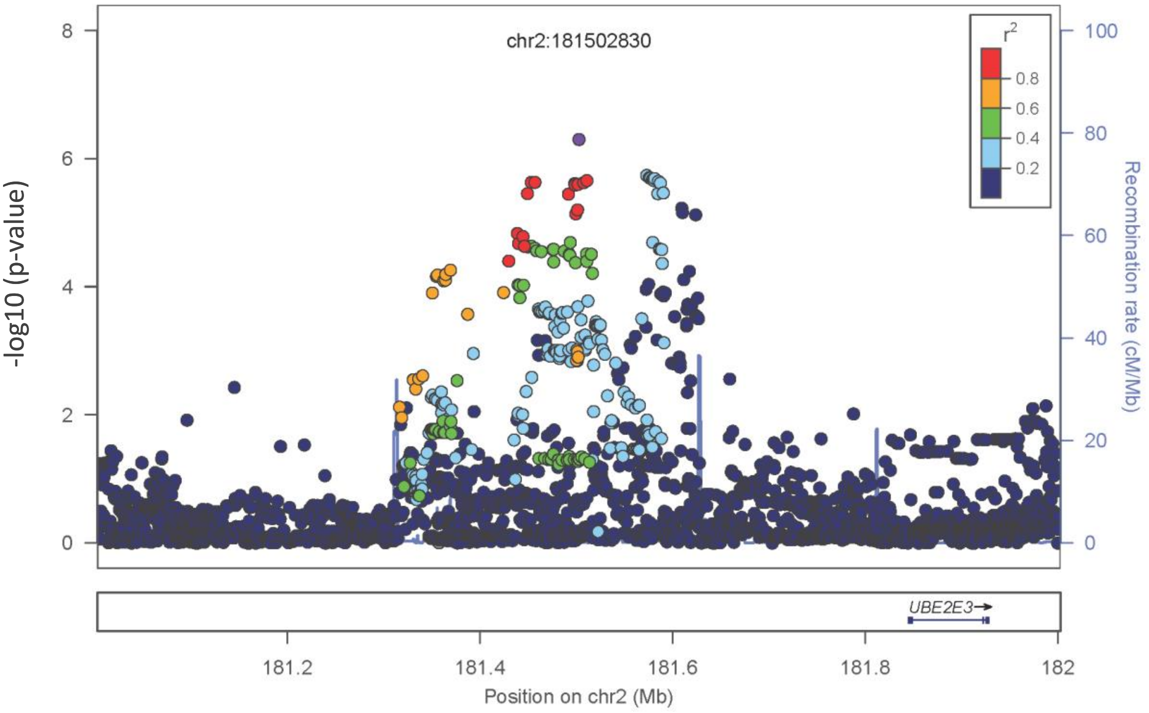

chr2:181502830
-log10 (p-value)
8
6
4
2
0
r2
0.8
0.6
0.4
0.2
100
80
60
40
20
0
Recombination rate (cM/Mb)
UBE2E3
181.2
181.4
181.6
181.8
182
Position on chr2 (Mb)

(c)

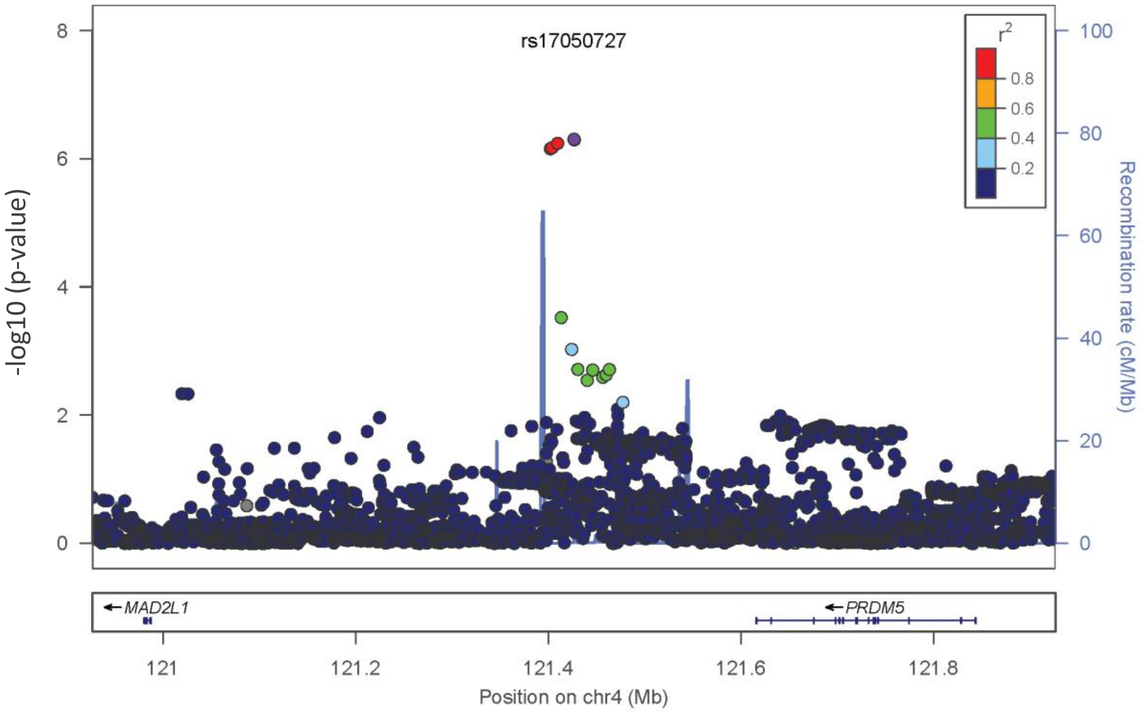

rs17050727
-log10 (p-value)
8
6
4
2
0
r2
0.8
0.6
0.4
0.2
100
80
60
40
20
0
Recombination rate (cM/Mb)
MAD2L1
PRDM5
121
121.2
121.4
121.6
121.8
Position on chr4 (Mb)

(d)

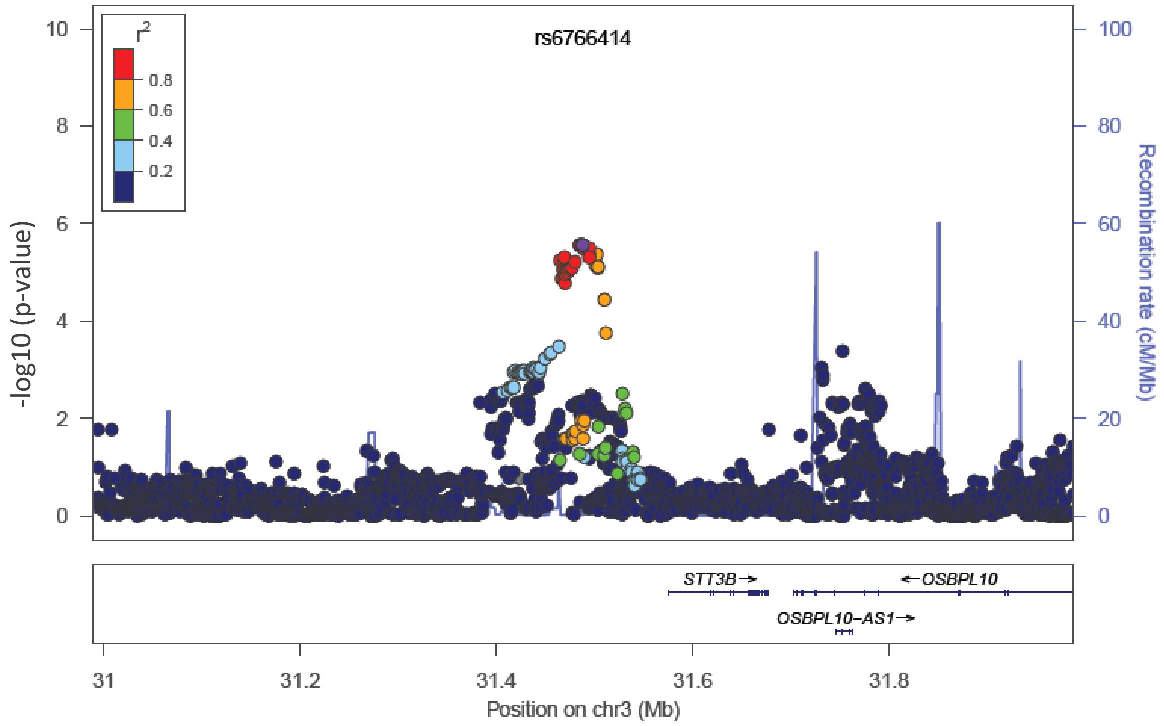
rs6766414
r2
0.8
0.6
0.4
0.2
-log10 (p-value)
Recombination rate (cM/Mb)
STT3B
OSBPL10
OSBPL10-AS1
Position on chr3 (Mb)

(e)

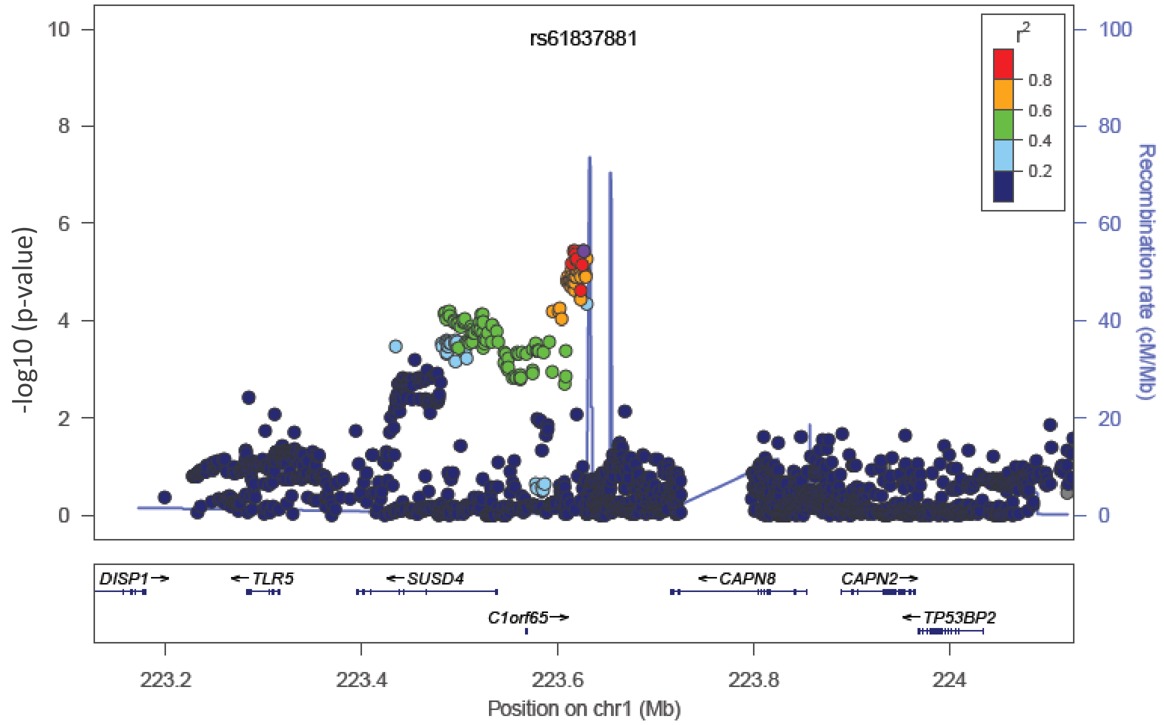
rs61837881
r2
0.8
0.6
0.4
0.2
-log10 (p-value)
Recombination rate (cM/Mb)
DISP1
TLR5
SUSD4
C1orf65
CAPN8
CAPN2
TP53BP2
Position on chr1 (Mb)

(f)

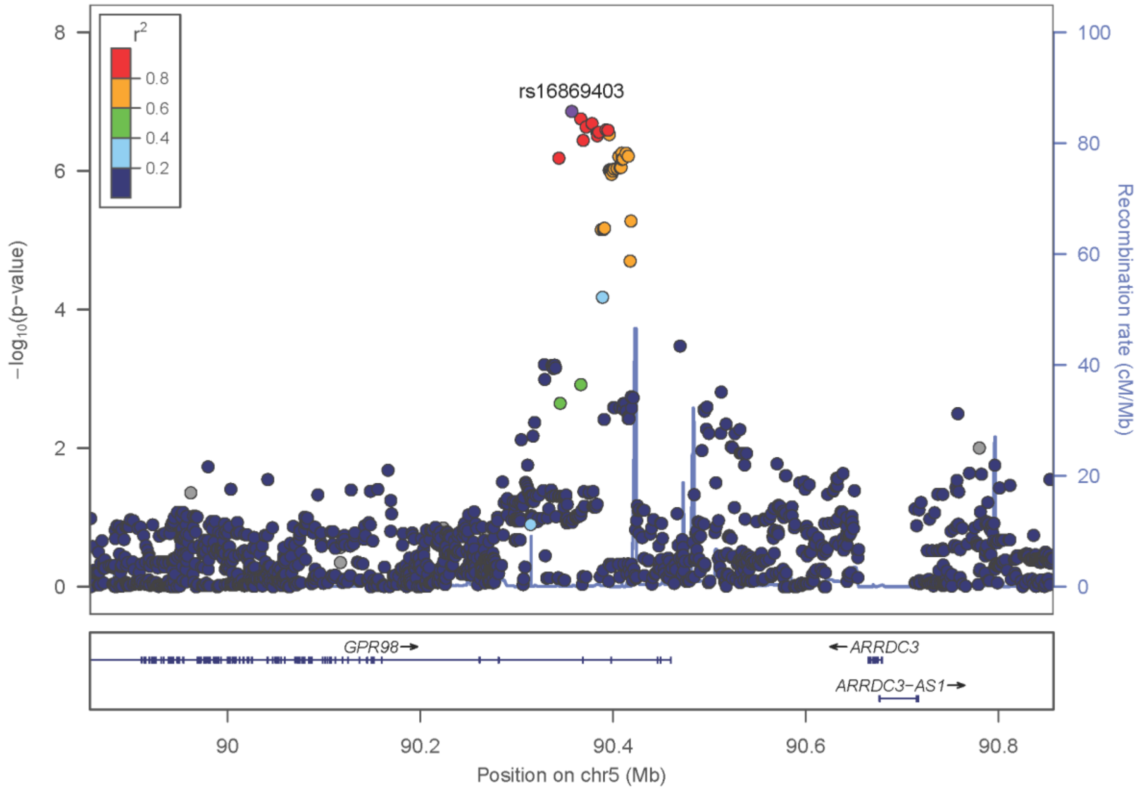
r²
0.8
0.6
0.4
0.2
rs16869403
8
6
4
2
0
−log10(p−value)
100
80
60
40
20
0
Recombination rate (cM/Mb)
GPR98→
←ARRDC3
ARRDC3−AS1→
90
90.2
90.4
90.6
90.8
Position on chr5 (Mb)

Figure S3. QQ and Manhattan plots for secondary analyses of hip OA stratified by anatomic pattern of joint involvement and by bone remodelling response vs. population-based controls and of all hip OA cases vs. population-based controls. (a) GWAS of hip OA cases with superior joint space narrowing vs. population-based controls; (b) GWAS of hip OA cases with axial/medial joint space narrowing vs. population-based controls; (c) GWAS of hip OA cases with hypertrophic bone response vs. population-based controls; (d) GWAS of hip OA cases with atrophic bone response vs. population-based controls; (e) GWAS of hip OA cases vs. population-based controls.

(a)

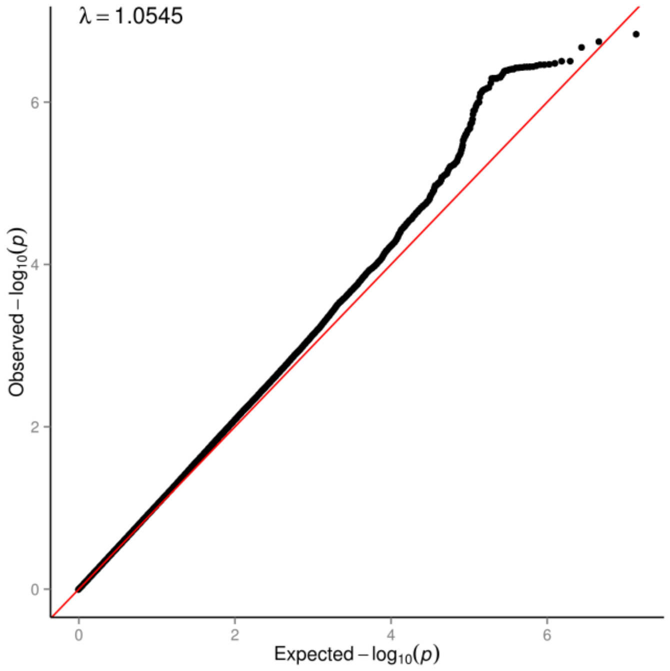

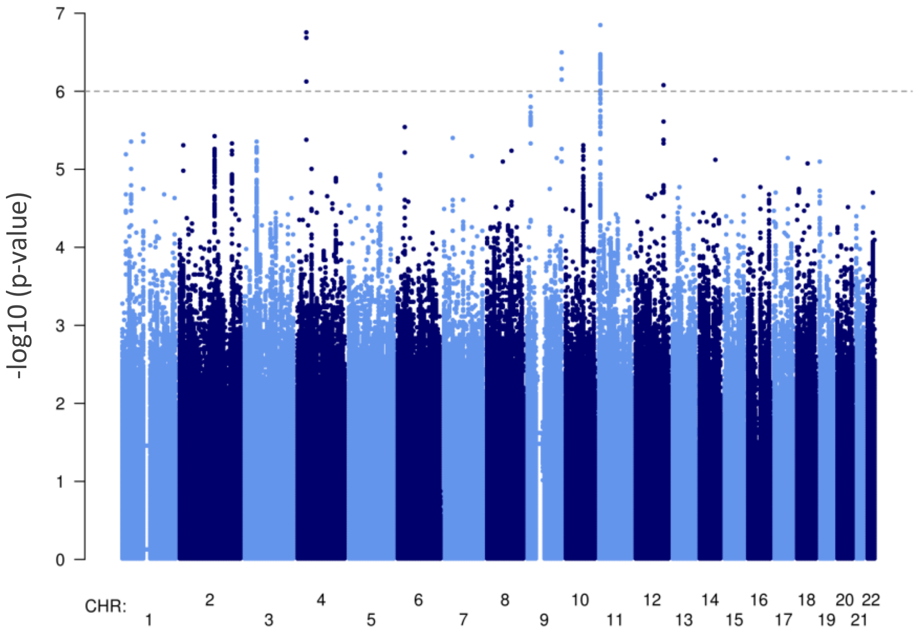

(b)

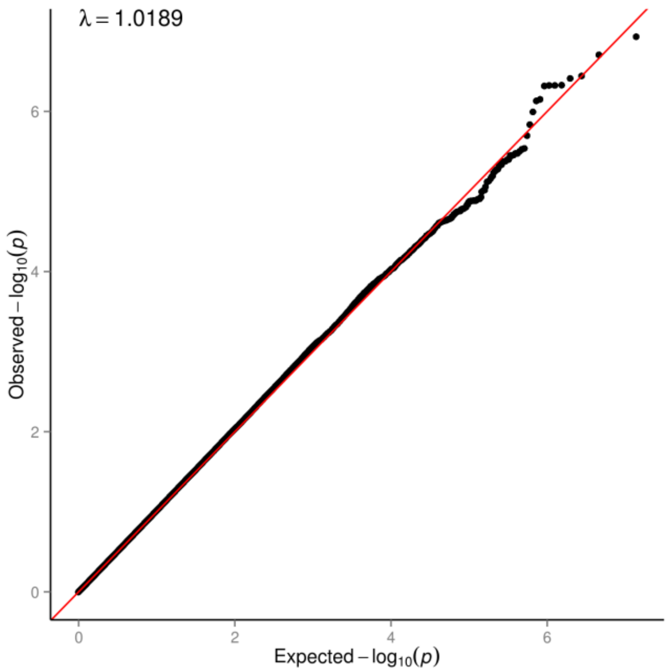
λ = 1.0189
Observed − log10(p)
Expected − log10(p)
0
2
4
6
0
2
4
6

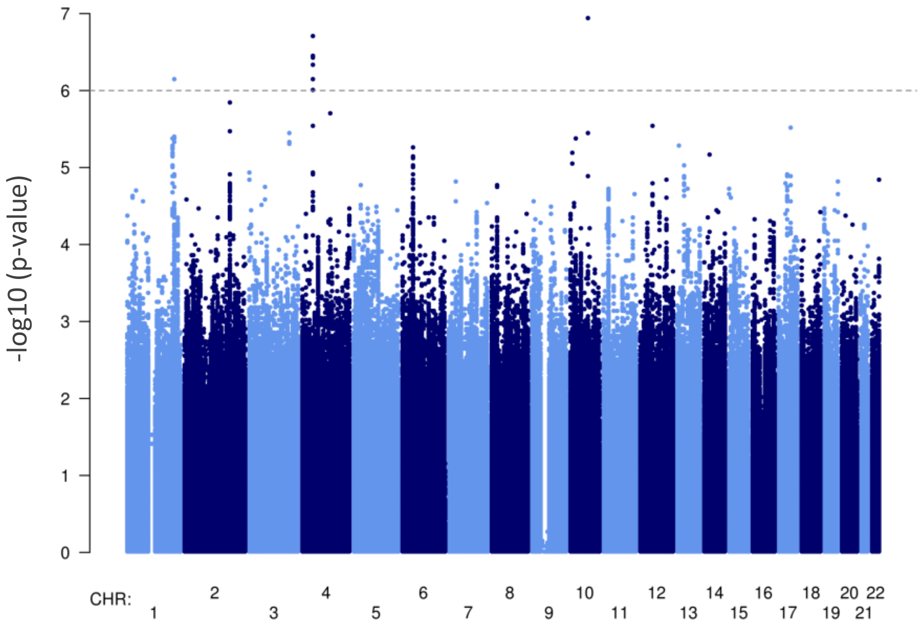
-log10 (p-value)
7
6
5
4
3
2
1
0
CHR:
1
2
3
4
5
6
7
8
9
10
11
12
13
14
15
16
17
18
19
20
21
22

(c)

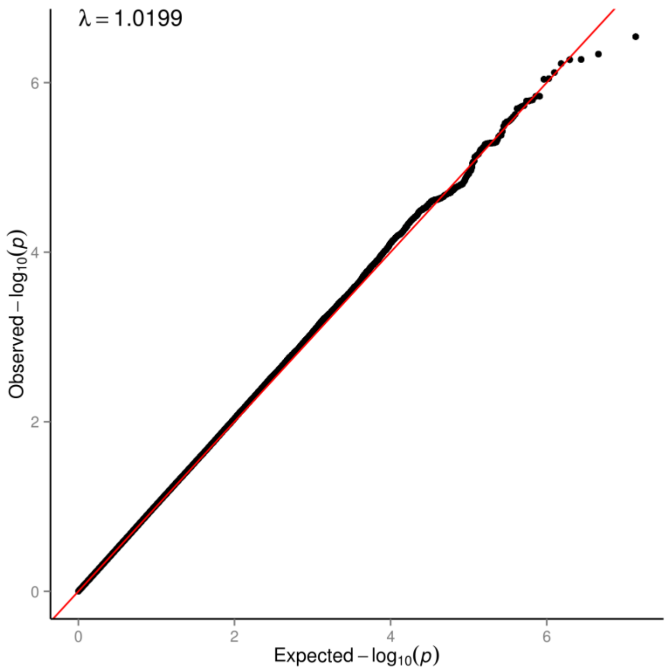
λ = 1.0199
Observed − log10(p)
Expected − log10(p)
0
2
4
6

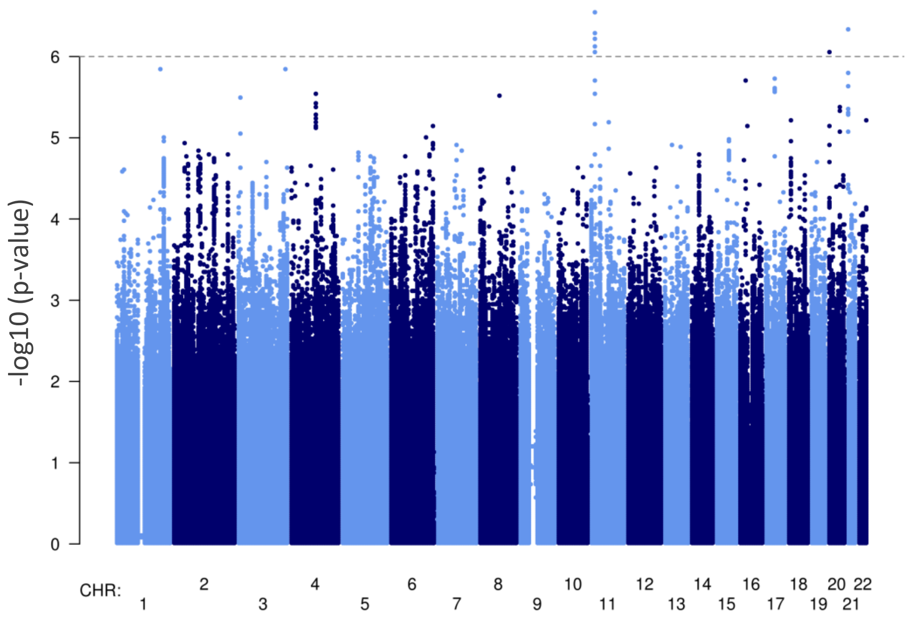
-log10 (p-value)
CHR:
1 2 3 4 5 6 7 8 9 10 11 12 13 14 15 16 17 18 19 20 21 22

(d)

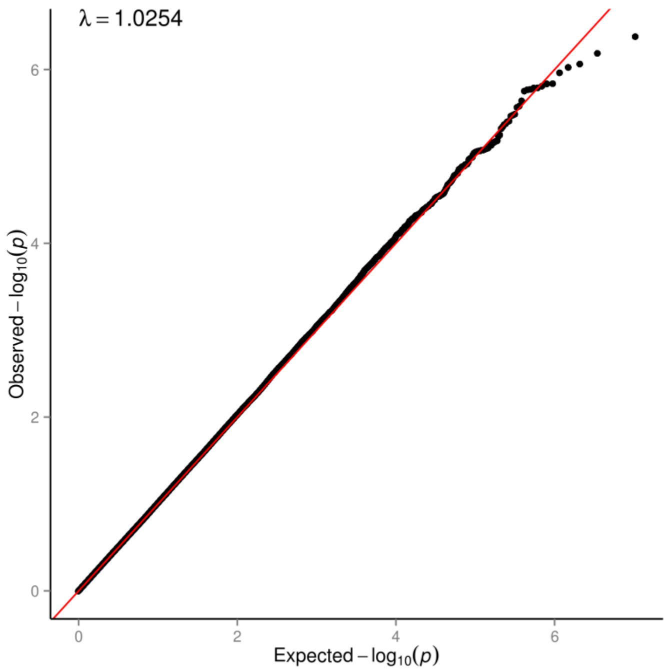
λ = 1.0254
Observed − log10(p)
Expected − log10(p)
0
2
4
6

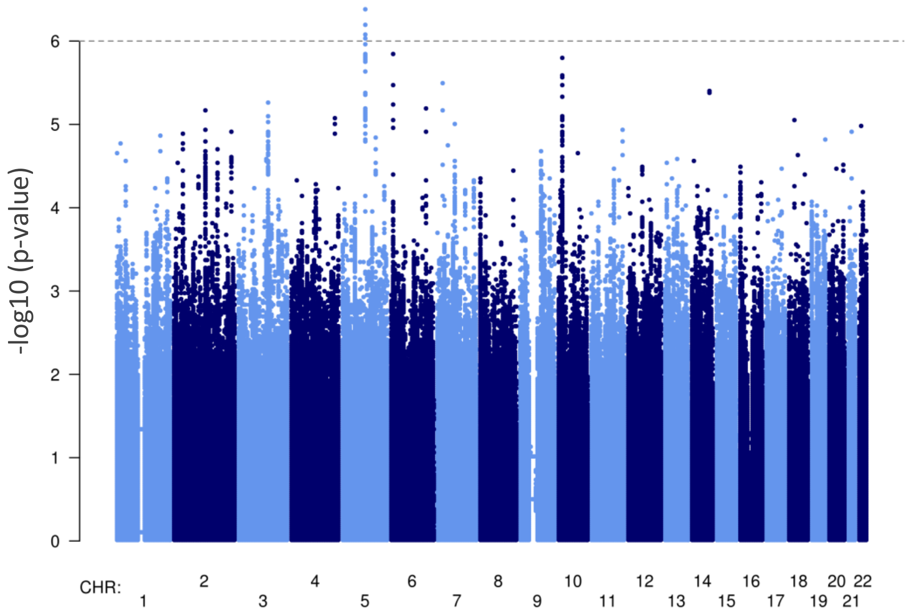
-log10 (p-value)
CHR:
1 2 3 4 5 6 7 8 9 10 11 12 13 14 15 16 17 18 19 20 21 22

(e)

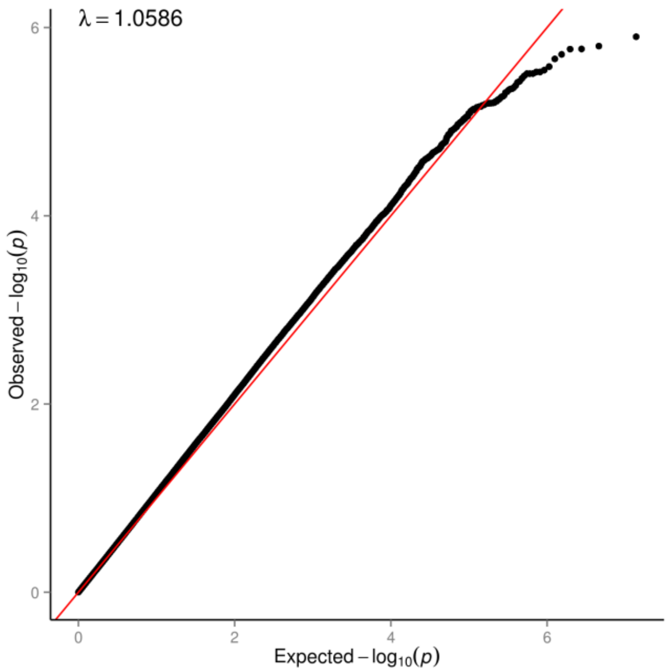
λ = 1.0586
Observed − log10(p)
Expected − log10(p)

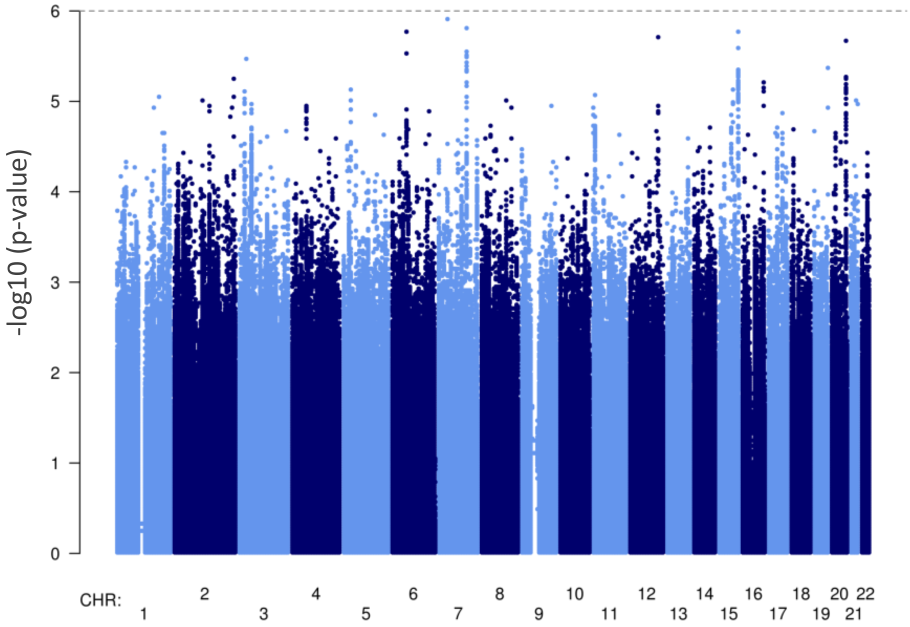
-log10 (p-value)
CHR:
1 2 3 4 5 6 7 8 9 10 11 12 13 14 15 16 17 18 19 20 21 22

SUPPLEMENTARY TABLES

Table S1. Summary statistics of the associated variants across relevant strata. The stratum in which the strength of association was greater for each variant is shown in bold.

SNP, single nucleotide polymorphism; EA, effect allele; EAF, effect allele frequency; Q_p, Cochran's heterogeneity statistic's p-value; I^2, heterogeneity index; OA, osteoarthritis; JSN, joint space narrowing.

SNP	EA	Analysis	Definition of cases	Definition of controls	N case/control	EAF case/control	OR[95%CI]	P	Q_p	I^2(%)
**rs754106**	**T**	**Meta-analysis**	**Hip OA with superior JSN**	**Hip OA with non-superior JSN**	**1469/649**	**0.51/0.60**	**0.70[0.61-0.80]**	**1.49E-07**	**0.19**	**41**
		GWAS set 1	Hip OA with superior JSN	Hip OA with non-superior JSN	1265/552	0.52/0.60	0.72[0.62-0.83]	7.95E-06		
		GWAS set 2	Hip OA with superior JSN	Hip OA with non-superior JSN	204/97	0.49/0.63	0.55[0.38-0.80]	1.37E-03		
		GWAS	Hip OA with superior JSN	Population-based	1265/6500	0.52/0.54	0.91[0.84-0.99]	3.43E-02		
		Meta-analysis	Hip OA with axial/medial JSN	Hip OA with non-axial/medial JSN	501/1617	0.61/0.52	1.47[1.27-1.70]	3.66E-07	0.34	0
		GWAS set 1	Hip OA with axial/medial JSN	Hip OA with non-axial/medial JSN	425/1392	0.61/0.52	1.43[1.22-1.67]	9.63E-06		
		GWAS set 2	Hip OA with axial/medial JSN	Hip OA with non-axial/medial JSN	76/225	0.63/0.50	1.76[1.18-2.63]	4.57E-03		
		GWAS	Hip OA with axial/medial JSN	Population-based	425/6500	0.61/0.54	1.32[1.14-1.52]	1.39E-04		
		GWAS	Hip OA	Population-based	1817/6500	0.54/0.54	1.01[0.93-1.08]	8.94E-01		
**rs73023563**	**T**	**Meta-analysis**	**Hip OA with superior JSN**	**Hip OA with non-superior JSN**	**1469/649**	**0.21/0.14**	**1.59[1.33-1.91]**	**6.59E-07**	**0.56**	**0**
		GWAS set 1	Hip OA with superior JSN	Hip OA with non-superior JSN	1265/552	0.21/0.14	1.63[1.33-1.99]	6.22E-07		
		GWAS set 2	Hip OA with superior JSN	Hip OA with non-superior JSN	204/97	0.21/0.17	1.40[0.87-2.25]	1.60E-01		
		GWAS	Hip OA with superior JSN	Population-based	1265/6500	0.21/0.20	1.07[0.97-1.19]	1.93E-01		
		Meta-analysis	Hip OA with axial/medial JSN	Hip OA with non-axial/medial JSN	501/1617	0.14/0.20	0.61[0.50-0.75]	2.03E-06	0.83	0
		GWAS set 1	Hip OA with axial/medial JSN	Hip OA with non-axial/medial JSN	425/1392	0.13/0.20	0.60[0.48-0.75]	3.13E-06		
		GWAS set 2	Hip OA with axial/medial JSN	Hip OA with non-axial/medial JSN	76/225	0.15/0.21	0.64[0.38-1.09]	9.19E-02		
		GWAS	Hip OA with axial/medial JSN	Population-based	425/6500	0.13/0.20	0.63[0.52-0.78]	3.45E-06		
		GWAS	Hip OA	Population-based	1817/6500	0.19/0.20	0.94[0.86-1.03]	2.06E-01		
**rs17050727**	**T**	**Meta-analysis**	**Hip OA with axial/medial JSN**	**Hip OA with non-axial/medial JSN**	**501/1617**	**0.18/0.12**	**1.65[1.35-2.01]**	**6.87E-07**	**0.97**	**0**
		GWAS set 1	Hip OA with axial/medial JSN	Hip OA with non-axial/medial JSN	425/1392	0.18/0.12	1.65[1.33-2.05]	7.13E-06		
		GWAS set 2	Hip OA with axial/medial JSN	Hip OA with non-axial/medial JSN	76/225	0.19/0.12	1.64[1.00-2.69]	5.52E-02		
		GWAS	Hip OA with axial/medial JSN	Population-based	425/6500	0.18/0.14	1.43[1.19-1.72]	2.01E-04		
		Meta-analysis	Hip OA with superior JSN	Hip OA with non-superior JSN	1469/649	0.12/0.17	0.67[0.56-0.81]	3.24E-05	0.58	0
		GWAS set 1	Hip OA with superior JSN	Hip OA with non-superior JSN	1265/552	0.12/0.17	0.66[0.53-0.80]	6.21E-05		
		GWAS set 2	Hip OA with superior JSN	Hip OA with non-superior JSN	204/97	0.13/0.16	0.76[0.47-1.23]	2.66E-01		

		GWAS	Hip OA with superior JSN	Population-based	1265/6500	0.12/0.14	0.87[0.76-0.99]	3.89E-02		
		GWAS	Hip OA	Population-based	1817/6500	0.14/0.14	1.00[0.90-1.12]	9.78E-01		
**rs6766414**	**G**	**Meta-analysis**	**Hip OA with hypertrophic response**	**Hip OA with non-hypertrophic response**	**637/1481**	**0.29/0.22**	**1.45[1.24-1.69]**	**3.13E-06**	**0.52**	**0**
		GWAS set 1	Hip OA with hypertrophic response	Hip OA with non-hypertrophic response	531/1286	0.28/0.22	1.42[1.20-1.68]	5.84E-05		
		GWAS set 2	Hip OA with hypertrophic response	Hip OA with non-hypertrophic response	106/195	0.31/0.22	1.63[1.10-2.42]	1.48E-02		
		GWAS	Hip OA with hypertrophic response	Population-based	531/6500	0.28/0.24	1.25[1.09-1.44]	1.74E-03		
		GWAS set 1	Hip OA with atrophic response	Hip OA with non-atrophic response	267/1550	0.22/0.24	0.86[0.68-1.08]	1.79E-01		
		GWAS	Hip OA with atrophic response	Population-based	267/6500	0.22/0.24	0.88[0.72-1.09]	2.44E-01		
		GWAS	Hip OA	Population-based	1817/6500	0.24/0.24	1.00[0.92-1.09]	9.76E-01		
**rs61837881**	**C**	**Meta-analysis**	**Hip OA with hypertrophic response**	**Hip OA with non-hypertrophic response**	**637/1481**	**0.29/0.22**	**1.44[1.23-1.68]**	**4.56E-06**	**0.27**	**16**
		GWAS set 1	Hip OA with hypertrophic response	Hip OA with non-hypertrophic response	531/1286	0.29/0.23	1.39[1.18-1.64]	1.10E-04		
		GWAS set 2	Hip OA with hypertrophic response	Hip OA with non-hypertrophic response	106/195	0.29/0.20	1.80[1.17-2.76]	7.14E-03		
		GWAS	Hip OA with hypertrophic response	Population-based	531/6500	0.29/0.23	1.33[1.15-1.53]	1.28E-04		
		GWAS set 1	Hip OA with atrophic response	Hip OA with non-atrophic response	267/1550	0.24/0.24	0.96[0.77-1.19]	6.93E-01		
		GWAS	Hip OA with atrophic response	Population-based	267/6500	0.24/0.23	1.02[0.82-1.25]	8.79E-01		
		GWAS	Hip OA	Population-based	1817/6500	0.24/0.23	1.06[0.97-1.15]	2.28E-01		
**rs16869403**	**G**	**GWAS set 1**	**Hip OA with atrophic response**	**Hip OA with non-atrophic response**	**267/1550**	**0.17/0.09**	**2.11[1.61-2.75]**	**1.37E-07**		
		GWAS	Hip OA with atrophic response	Population-based	267/6500	0.17/0.10	1.93[1.52-2.46]	4.14E-07		
		Meta-analysis	Hip OA with hypertrophic response	Hip OA with non-hypertrophic response	637/1481	0.09/0.10	0.86[0.69-1.08]	2.08E-01	0.63	0
		GWAS set 1	Hip OA with hypertrophic response	Hip OA with non-hypertrophic response	531/1286	0.09/0.10	0.84[0.65-1.08]	1.75E-01		
		GWAS set 2	Hip OA with hypertrophic response	Hip OA with non-hypertrophic response	106/195	0.10/0.10	0.97[0.57-1.64]	9.09E-01		
		GWAS	Hip OA with hypertrophic	Population-based	531/6500	0.09/0.10	0.93[0.74-1.16]	5.14E-01		

			response							
		GWAS	Hip OA	Population-based	1817/6500	0.10/0.10	1.05[0.93-1.19]	4.28E-01		

Table S2. Summary statistics of the associated variants before and after adjustment for sex, height and body mass index in the first set of arcOGEN cases.

SNP, single nucleotide polymorphism; JSN, joint space narrowing.

SNP	Cases Definition	Controls definition	N case/control	P	P_adjusted
rs754106	Hip OA with superior JSN	Hip OA with non-superior JSN	1234/525	4.10E-06	5.25E-05
rs754106	Hip OA with axial/medial JSN	Hip OA with non-axial/medial JSN	404/1355	1.05E-05	1.75E-04
rs73023563	Hip OA with superior JSN	Hip OA with non-superior JSN	1234/525	2.34E-07	1.80E-07
rs17050727	Hip OA with axial/medial JSN	Hip OA with non-axial/medial JSN	1234/525	4.35E-05	4.68E-05
rs73023563	Hip OA with axial/medial JSN	Hip OA with non-axial/medial JSN	404/1355	1.74E-06	1.14E-06
rs6766414	Hip OA with hypertrophic response	Hip OA with non-hypertrophic response	518/1241	2.90E-05	3.19E-05
rs61837881	Hip OA with hypertrophic response	Hip OA with non-hypertrophic response	518/1241	1.97E-04	2.37E-04

Table S3. Validation of top signals by Sequenom MassARRAY iPLEX Gold assay.

SNP, single nucleotide polymorphism; EA, effect allele; EAF, effect allele frequency; amvnam, hip osteoarthritis (OA) with axial/medial joint space narrowing vs. hip OA with non-axial/medial joint space narrowing; avna, hip OA with atrophic bone response vs. hip OA with non-atrophic response.

				Sequenom summary statistics				GWAS summary statistics					Concordance between Sequenom and GWAS	
**SNP**	**EA**	**Stratum**	**N case/control**	**OR [95%CI]**	**P**	**EAF cases**	**EAF controls**	**OR [95%CI]**	**P**	**EAF cases**	**EAF controls**	**Imputation score**	**% Genotype concordance**	**% Minor allele concordance**
rs754106	T	amvnam	413/1366	1.43 [1.21-1.68]	1.35×10^{-5}	0.393	0.479	1.42 [1.21-1.67]	1.37×10^{-5}	0.393	0.478	1	100	100
rs16869403	G	avna	262/1517	2.05 [1.57-2.68]	3.33×10^{-7}	0.170	0.092	2.11 [1.61-2.77]	2.36×10^{-7}	0.164	0.088	0.97	99	97

Table S4. Association summary statistics of established hip osteoarthritis loci across all strata examined.

SNP, single nucleotide polymorphism; EA, effect allele; EAF, effect allele frequency; JSN, joint space narrowing.

**Association summary statistics in discovery study**							**Association summary statistics in this study**				
**SNP**	**Nearest gene(s)**	**EA**	**Site**	**Sex**	**OR[95%CIs]**	**P**	**Definition of cases**	**Definition of controls**	**Analysis**	**OR[95%CI]**	**P**
rs12982744	*DOT1L*	C	Hip	Males	1.17[1.11-1.23]	7.8E-09	Hip OA	Population-based	GWAS	1.15[1.07-1.24]	3.06E-04
							Hip OA with axial/medial JSN	Population-based	GWAS	0.98[0.85-1.14]	8.33E-01
							Hip OA with axial/medial JSN	Hip OA with non-axial/medial JSN	GWAS set 1	0.81[0.69-0.95]	1.13E-02
							Hip OA with axial/medial JSN	Hip OA with non-axial/medial JSN	GWAS set 2	0.94[0.64-1.38]	7.62E-01
							Hip OA with superior JSN	Population-based	GWAS	1.22[1.12-1.34]	8.17E-06
							Hip OA with superior JSN	Hip OA with non-superior JSN	GWAS set 1	1.22[1.06-1.42]	7.58E-03
							Hip OA with superior JSN	Hip OA with non-superior JSN	GWAS set 2	1.02[0.71-1.45]	9.34E-01
							Hip OA with atrophic bone response	Population-based	GWAS	1.35[1.12-1.62]	1.39E-03
							Hip OA with atrophic bone response	Hip OA with non-atrophic response	GWAS	1.20[0.99-1.46]	6.33E-02
							Hip OA with hypertophic bone response	Population-based	GWAS	1.12[0.99-1.28]	7.62E-02
							Hip OA with hypertophic bone response	Hip OA with non-hypertophic response	GWAS set 1	0.97[0.83-1.12]	6.63E-01
							Hip OA with hypertophic bone response	Hip OA with non-hypertophic response	GWAS set 2	0.95[0.67-1.34]	7.51E-01
rs9350591	*FILIP1; SENP6*	T	Hip	Both	1.18[1.12-1.25]	2.4E-09	Hip OA	Population-based	GWAS	1.17[1.04-1.30]	7.01E-03
							Hip OA with axial/medial JSN	Population-based	GWAS	1.36[1.12-1.66]	3.11E-03
							Hip OA with axial/medial JSN	Hip OA with non-axial/medial JSN	GWAS set 1	1.23[0.98-1.53]	7.47E-02
							Hip OA with axial/medial JSN	Hip OA with non-axial/medial JSN	GWAS set 2	1.21[0.74-1.98]	4.42E-01
							Hip OA with superior JSN	Population-based	GWAS	1.05[0.92-1.20]	4.94E-01

							Hip OA with superior JSN	Hip OA with non-superior JSN	GWAS set 1	0.72[0.59-0.88]	1.73E-03
							Hip OA with superior JSN	Hip OA with non-superior JSN	GWAS set 2	0.85[0.53-1.34]	4.77E-01
							Hip OA with atrophic bone response	Population-based	GWAS	1.11[0.85-1.44]	4.57E-01
							Hip OA with atrophic bone response	Hip OA with non-atrophic response	GWAS	0.94[0.71-1.24]	6.60E-01
							Hip OA with hypertophic bone response	Population-based	GWAS	1.12[0.92-1.35]	2.64E-01
							Hip OA with hypertophic bone response	Hip OA with non-hypertophic response	GWAS set 1	0.94[0.76-1.16]	5.68E-01
							Hip OA with hypertophic bone response	Hip OA with non-hypertophic response	GWAS set 2	0.81[0.51-1.31]	3.88E-01
rs10948172	*SUPT3H*	G	Hip & knee	Males	1.14[1.09-1.20]	7.9E-08	Hip OA	Population-based	GWAS	1.13[1.04-1.22]	3.77E-03
							Hip OA with axial/medial JSN	Population-based	GWAS	1.24[1.07-1.44]	4.84E-03
							Hip OA with axial/medial JSN	Hip OA with non-axial/medial JSN	GWAS set 1	1.13[0.96-1.33]	1.42E-01
							Hip OA with axial/medial JSN	Hip OA with non-axial/medial JSN	GWAS set 2	1.01[0.69-1.50]	9.47E-01
							Hip OA with superior JSN	Population-based	GWAS	1.09[0.99-1.20]	7.49E-02
							Hip OA with superior JSN	Hip OA with non-superior JSN	GWAS set 1	0.90[0.77-1.04]	1.61E-01
							Hip OA with superior JSN	Hip OA with non-superior JSN	GWAS set 2	0.81[0.56-1.16]	2.41E-01
							Hip OA with atrophic bone response	Population-based	GWAS	1.14[0.94-1.37]	1.82E-01
							Hip OA with atrophic bone response	Hip OA with non-atrophic response	GWAS	1.01[0.83-1.23]	9.18E-01
							Hip OA with hypertophic bone response	Population-based	GWAS	1.18[1.03-1.35]	1.52E-02
							Hip OA with hypertophic bone response	Hip OA with non-hypertophic response	GWAS set 1	1.07[0.92-1.24]	3.84E-01
							Hip OA with hypertophic bone response	Hip OA with non-hypertophic response	GWAS set 2	0.98[0.68-1.40]	9.04E-01
rs11177	*GNL3*	A	Hip & knee	Both	1.12[1.08-1.16]	1.3E-10	Hip OA	Population-based	GWAS	1.17[1.08-1.25]	5.42E-05
							Hip OA with axial/medial JSN	Population-based	GWAS	1.01[0.88-1.16]	8.81E-01

							Hip OA with axial/medial JSN	Hip OA with non-axial/medial JSN	GWAS set 1	0.84[0.72-0.97]	2.16E-02
							Hip OA with axial/medial JSN	Hip OA with non-axial/medial JSN	GWAS set 2	0.90[0.62-1.31]	5.89E-01
							Hip OA with superior JSN	Population-based	GWAS	1.21[1.11-1.31]	1.97E-05
							Hip OA with superior JSN	Hip OA with non-superior JSN	GWAS set 1	1.11[0.97-1.28]	1.29E-01
							Hip OA with superior JSN	Hip OA with non-superior JSN	GWAS set 2	1.09[0.77-1.54]	6.39E-01
							Hip OA with atrophic bone response	Population-based	GWAS	1.06[0.89-1.27]	5.10E-01
							Hip OA with atrophic bone response	Hip OA with non-atrophic response	GWAS	0.90[0.75-1.08]	2.53E-01
							Hip OA with hypertophic bone response	Population-based	GWAS	1.09[0.96-1.24]	1.86E-01
							Hip OA with hypertophic bone response	Hip OA with non-hypertophic response	GWAS set 1	0.91[0.79-1.05]	1.99E-01
							Hip OA with hypertophic bone response	Hip OA with non-hypertophic response	GWAS set 2	1.31[0.93-1.84]	1.17E-01
rs4836732	*ASTN2*	C	Hip	Females	1.2[1.13-1.27]	6.1E-10	Hip OA	Population-based	GWAS	1.11[1.03-1.20]	4.66E-03
							Hip OA with axial/medial JSN	Population-based	GWAS	1.04[0.91-1.19]	5.75E-01
							Hip OA with axial/medial JSN	Hip OA with non-axial/medial JSN	GWAS set 1	0.92[0.79-1.07]	2.68E-01
							Hip OA with axial/medial JSN	Hip OA with non-axial/medial JSN	GWAS set 2	0.89[0.62-1.29]	5.45E-01
							Hip OA with superior JSN	Population-based	GWAS	1.13[1.04-1.23]	5.84E-03
							Hip OA with superior JSN	Hip OA with non-superior JSN	GWAS set 1	1.05[0.91-1.20]	5.36E-01
							Hip OA with superior JSN	Hip OA with non-superior JSN	GWAS set 2	1.07[0.76-1.50]	6.88E-01
							Hip OA with atrophic bone response	Population-based	GWAS	1.16[0.97-1.37]	1.01E-01
							Hip OA with atrophic bone response	Hip OA with non-atrophic response	GWAS	1.05[0.87-1.26]	6.28E-01
							Hip OA with hypertophic bone response	Population-based	GWAS	1.11[0.98-1.25]	1.17E-01
							Hip OA with hypertophic bone response	Hip OA with non-hypertophic response	GWAS set 1	0.99[0.86-1.14]	9.03E-01

							Hip OA with hypertophic bone response	Hip OA with non-hypertophic response	GWAS set 2	1.18[0.84-1.64]	3.39E-01
rs6094710	*NCOA3*	A	Hip	Both	1.28[1.18-1.39]	7.9E-09	Hip OA	Population-based	GWAS	1.26[1.04-1.54]	2.32E-02
							Hip OA with axial/medial JSN	Population-based	GWAS	1.50[1.07-2.10]	2.49E-02
							Hip OA with axial/medial JSN	Hip OA with non-axial/medial JSN	GWAS set 1	1.28[0.87-1.87]	2.23E-01
							Hip OA with axial/medial JSN	Hip OA with non-axial/medial JSN	GWAS set 2	0.67[0.25-1.78]	4.01E-01
							Hip OA with superior JSN	Population-based	GWAS	1.19[0.94-1.50]	1.49E-01
							Hip OA with superior JSN	Hip OA with non-superior JSN	GWAS set 1	0.83[0.58-1.19]	3.16E-01
							Hip OA with superior JSN	Hip OA with non-superior JSN	GWAS set 2	1.68[0.67-4.19]	2.46E-01
							Hip OA with atrophic bone response	Population-based	GWAS	1.33[0.85-2.09]	2.34E-01
							Hip OA with atrophic bone response	Hip OA with non-atrophic response	GWAS	1.05[0.65-1.72]	8.34E-01
							Hip OA with hypertophic bone response	Population-based	GWAS	1.57[1.16-2.13]	6.58E-03
							Hip OA with hypertophic bone response	Hip OA with non-hypertophic response	GWAS set 1	1.36[0.95-1.96]	9.93E-02
							Hip OA with hypertophic bone response	Hip OA with non-hypertophic response	GWAS set 2	1.27[0.58-2.76]	5.49E-01
rs835487	*CHST11*	G	Hip	Both	1.13[1.09-1.18]	1.6E-08	Hip OA	Population-based	GWAS	1.17[1.09-1.27]	4.28E-05
							Hip OA with axial/medial JSN	Population-based	GWAS	1.09[0.94-1.26]	2.37E-01
							Hip OA with axial/medial JSN	Hip OA with non-axial/medial JSN	GWAS set 1	0.91[0.77-1.07]	2.38E-01
							Hip OA with axial/medial JSN	Hip OA with non-axial/medial JSN	GWAS set 2	1.50[1.03-2.20]	3.62E-02
							Hip OA with superior JSN	Population-based	GWAS	1.19[1.09-1.30]	1.73E-04
							Hip OA with superior JSN	Hip OA with non-superior JSN	GWAS set 1	1.03[0.89-1.20]	6.56E-01
							Hip OA with superior JSN	Hip OA with non-superior JSN	GWAS set 2	0.77[0.54-1.10]	1.45E-01
							Hip OA with atrophic bone response	Population-based	GWAS	1.26[1.05-1.50]	1.22E-02

							Hip OA with atrophic bone response	Hip OA with non-atrophic response	GWAS	1.09[0.90-1.32]	3.79E-01
							Hip OA with hypertophic bone response	Population-based	GWAS	1.18[1.03-1.34]	1.40E-02
							Hip OA with hypertophic bone response	Hip OA with non-hypertophic response	GWAS set 1	1.01[0.86-1.17]	9.39E-01
							Hip OA with hypertophic bone response	Hip OA with non-hypertophic response	GWAS set 2	1.20[0.85-1.70]	3.10E-01
rs8044769	*FTO*	C	Hip & knee	Females	1.11[1.07-1.15]	6.9E-08	Hip OA	Population-based	GWAS	1.13[1.05-1.22]	8.92E-04
							Hip OA with axial/medial JSN	Population-based	GWAS	1.17[1.02-1.34]	2.89E-02
							Hip OA with axial/medial JSN	Hip OA with non-axial/medial JSN	GWAS set 1	1.04[0.89-1.21]	6.20E-01
							Hip OA with axial/medial JSN	Hip OA with non-axial/medial JSN	GWAS set 2	1.40[0.95-2.06]	9.02E-02
							Hip OA with superior JSN	Population-based	GWAS	1.12[1.03-1.22]	8.36E-03
							Hip OA with superior JSN	Hip OA with non-superior JSN	GWAS set 1	0.97[0.84-1.12]	6.48E-01
							Hip OA with superior JSN	Hip OA with non-superior JSN	GWAS set 2	0.75[0.52-1.08]	1.17E-01
							Hip OA with atrophic bone response	Population-based	GWAS	1.12[0.94-1.33]	2.18E-01
							Hip OA with atrophic bone response	Hip OA with non-atrophic response	GWAS	0.98[0.82-1.18]	8.38E-01
							Hip OA with hypertophic bone response	Population-based	GWAS	1.16[1.02-1.32]	2.11E-02
							Hip OA with hypertophic bone response	Hip OA with non-hypertophic response	GWAS set 1	1.03[0.89-1.19]	6.62E-01
							Hip OA with hypertophic bone response	Hip OA with non-hypertophic response	GWAS set 2	1.13[0.80-1.59]	5.03E-01
rs10492367	*KLHDC5; PTHLH*	T	Hip	Both	1.14[1.09-1.20]	1.5E-08	Hip OA	Population-based	GWAS	1.12[1.02-1.23]	1.87E-02
							Hip OA with axial/medial JSN	Population-based	GWAS	1.11[0.93-1.32]	2.54E-01
							Hip OA with axial/medial JSN	Hip OA with non-axial/medial JSN	GWAS set 1	0.99[0.81-1.20]	8.99E-01
							Hip OA with axial/medial JSN	Hip OA with non-axial/medial JSN	GWAS set 2	0.95[0.19-4.82]	9.48E-01
							Hip OA with superior JSN	Population-based	GWAS	1.12[1.01-1.25]	3.52E-02

							Hip OA with superior JSN	Hip OA with non-superior JSN	GWAS set 1	1.01[0.85-1.21]	8.80E-01
							Hip OA with superior JSN	Hip OA with non-superior JSN	GWAS set 2	0.85[0.20-3.49]	8.19E-01
							Hip OA with atrophic bone response	Population-based	GWAS	1.10[0.89-1.37]	3.79E-01
							Hip OA with atrophic bone response	Hip OA with non-atrophic response	GWAS	0.98[0.78-1.24]	8.95E-01
							Hip OA with hypertophic bone response	Population-based	GWAS	1.19[1.02-1.39]	3.13E-02
							Hip OA with hypertophic bone response	Hip OA with non-hypertophic response	GWAS set 1	1.09[0.91-1.30]	3.41E-01
							Hip OA with hypertophic bone response	Hip OA with non-hypertophic response	GWAS set 2	1.91[0.46-7.91]	3.67E-01
rs11842874	MCF2L	A	Knee & Hip	Both	1.17[1.11-1.23]	2.1E-08	Hip OA	Population-based	GWAS	1.07[0.93-1.23]	3.66E-01
							Hip OA with axial/medial JSN	Population-based	GWAS	1.12[0.86-1.47]	3.96E-01
							Hip OA with axial/medial JSN	Hip OA with non-axial/medial JSN	GWAS set 1	1.07[0.79-1.45]	6.56E-01
							Hip OA with axial/medial JSN	Hip OA with non-axial/medial JSN	GWAS set 2	1.57[0.66-3.74]	2.86E-01
							Hip OA with superior JSN	Population-based	GWAS	1.07[0.91-1.26]	4.14E-01
							Hip OA with superior JSN	Hip OA with non-superior JSN	GWAS set 1	1.01[0.77-1.33]	9.38E-01
							Hip OA with superior JSN	Hip OA with non-superior JSN	GWAS set 2	1.27[0.62-2.57]	5.19E-01
							Hip OA with atrophic bone response	Population-based	GWAS	1.23[0.86-1.75]	2.38E-01
							Hip OA with atrophic bone response	Hip OA with non-atrophic response	GWAS	1.18[0.81-1.73]	3.70E-01
							Hip OA with hypertophic bone response	Population-based	GWAS	1.09[0.86-1.39]	4.82E-01
							Hip OA with hypertophic bone response	Hip OA with non-hypertophic response	GWAS set 1	1.03[0.78-1.36]	8.28E-01
							Hip OA with hypertophic bone response	Hip OA with non-hypertophic response	GWAS set 2	0.81[0.40-1.63]	5.59E-01
